# Morphodynamics of the Actin-Rich Cytoskeleton in *Entamoeba histolytica*

**DOI:** 10.3389/fcimb.2018.00179

**Published:** 2018-05-29

**Authors:** Maria Manich, Nora Hernandez-Cuevas, Juan D. Ospina-Villa, Sylvie Syan, Laurence A. Marchat, Jean-Christophe Olivo-Marin, Nancy Guillén

**Affiliations:** ^1^BioImaging Unit, Institut Pasteur, Paris, France; ^2^Cell Biology of Parasitism Unit, Institut Pasteur, Paris, France; ^3^Instituto Politécnico Nacional, Escuela Nacional de Medicina y Homeopatía, Mexico City, Mexico; ^4^Centre National de la Recherche Scientifique, CNRS-ERL9195, Paris, France

**Keywords:** *Entamoeba*, actin, macropinosome, HaloTag, Arp2/3

## Abstract

*Entamoeba histolytica* is the anaerobic protozoan parasite responsible for human amoebiasis, the third most deadly parasitic disease worldwide. This highly motile eukaryotic cell invades human tissues and constitutes an excellent experimental model of cell motility and cell shape deformation. The absence of extranuclear microtubules in *Entamoeba histolytica* means that the actin-rich cytoskeleton takes on a crucial role in not only amoebic motility but also other processes sustaining pathogenesis, such as the phagocytosis of human cells and the parasite's resistance of host immune responses. Actin is highly conserved among eukaryotes, although diverse isoforms exist in almost all organisms studied to date. However, *E. histolytica* has a single actin protein, the structure of which differs significantly from those of its human homologs. Here, we studied the expression, structure and dynamics of actin in *E. histolytica*. We used molecular and cellular approaches to evaluate actin gene expression during intestinal invasion by *E. histolytica* trophozoites. Based on a three-dimensional structural bioinformatics analysis, we characterized protein domains differences between amoebic actin and human actin. Fine-tuned molecular dynamics simulations enabled us to examine protein motion and refine the three-dimensional structures of both actins, including elements potentially accounting for differences changes in the affinity properties of amoebic actin and deoxyribonuclease I. The dynamic, multifunctional nature of the amoebic cytoskeleton prompted us to examine the pleiotropic forms of actin structures within live *E. histolytica* cells; we observed the cortical cytoskeleton, stress fibers, “dot-like” structures, adhesion plates, and macropinosomes. In line with these data, a proteomics study of actin-binding proteins highlighted the Arp2/3 protein complex as a crucial element for the development of macropinosomes and adhesion plaques.

## Introduction

Actin is a fundamental component of the cytoskeleton. It is able to form robust cellular scaffolds (called microfilaments) that underpin the vast majority of motile events in eukaryotic cells, including changes in cell shape and in the morphology of the endomembrane system (Svitkina, 2018). The actin-rich cytoskeleton's ability to fulfill all these essential cellular functions depends on the precise spatiotemporal control of microfilament formation and turnover. Microfilament polymerization and depolymerization are tightly regulated by (i) the presence of diverse isoforms of actin in the same organism (there are three in humans, for example), and (ii) the existence of more than a hundred proteins associated with G-actin (monomeric actin) or F-actin and that regulate microfilament assembly or disassembly (for a review, see Pollard, 2016). These actin-binding proteins (ABPs) use subtle mechanisms of action to control microfilament organization in various networks. For instance, β-actin is the most strongly expressed of the three actin isoforms in mammalian cells, followed by γ-actin. The isoforms' intracellular localizations and functions differ, since β-actin is enriched in the frontal cell lamellipodia (forming dendritic scaffolds), while γ-actin is mostly present in actin arcs and/or stress fiber structures (mainly involved in the cell's adhesive properties). Furthermore, β-actin and γ-actin's functions require interactions with distinct ABPs (for a recent review, see Skruber et al., 2018).

In contrast to mammals, several unicellular organisms have a single actin protein that constitutes all the actin-rich cytoskeletal structures required for life. Here, we focused on *Entamoeba histolytica*, the protozoan parasite responsible for human amoebiasis. This infectious disease occurs at high incidence in large populations with limited modern sanitation systems. The infestation arises after ingestion of cysts contaminating water and food. Upon de-cystation, a vegetative cell, the trophozoite, is formed that colonizes the intestine or becomes invasive destroying the tissue during the disease process (Marie and Petri, 2014). *Entamoeba histolytica*'s invasive behavior relies on three main activities: motility, adhesion, and cell lysis/toxicity. In this context, the cytoskeleton is responsible for changes in cell shape and other pivotal processes, including motility, phagocytosis of human cells, and parasite-substrate interactions (Guillén, 1993). *E. histolytica* is a highly motile single cell that can rapidly alter its shape (Dufour et al., 2015). The dynamic reorganization of the cytoskeleton is crucial for all these processes—highlighting its central role in amoebic pathogenesis.

The actin-rich cytoskeleton is the major skeletal component in *E. histolytica* because its microtubules are solely intranuclear (Vayssié et al., 2004) and intermediate filaments are absent (Clark et al., 2007). In contrast to other eukaryotes (including other amoebae like *Dictyostelium discoideum*) *E. histolytica* has a single actin protein only. The initial evidence for the existence of actin in *E. histolytica* (referred to here as EhActin) was obtained by immunostaining with an antibody against human actin (HsActin) (Kettis et al., 1977). The purified actin protein was unable to bind to DNAse I, in contrast to the majority of known actins (Lazarides and Lindberg, 1974; Gadasi, 1982; Meza et al., 1983). In *E. histolytica*, cell displacement and other cell functions are correlated with the presence of various actin-enriched structures (Bailey et al., 1985; Talamás-Rohana and Meza, 1988; Dufour et al., 2015; Emmanuel et al., 2015). Very few of the many ABPs known to participate in the structural dynamics of actin filaments have been identified in *E. histolytica* (Meza et al., 2006; Hon et al., 2010). However, a few have been experimentally confirmed: ARPC1, a subunit of the Arp2/3 complex that participates in actin nucleation and filament dendritic organization (Babuta et al., 2015), and is involved in amoebic phagocytosis; formin, which stabilizes microfilaments (Majumder and Lohia, 2008); filamin (ABP-120), which organizes microfilaments into orthogonal networks (Vargas et al., 1996; Díaz-Valencia et al., 2007); and profilin, the G-actin-sequestering protein (Binder et al., 1995). Furthermore, coactosin binds and stabilizes F- actin and regulates microfilaments in *E. histolytica* (Kumar et al., 2014). Actin-binding protein 16 (a member of the actin depolymerizing factor/cofilin family) is necessary for *E. histolytica* motility (de la Cruz et al., 2014), whereas the F-actin-binding protein NCABP166 translocates into the nucleus and participates in phagocytosis and cell motility (Campos-Parra et al., 2010; Uribe et al., 2012). Due to the presence of a single actin protein in *E. histolytica*, we hypothesized that the actin-rich cytoskeleton interacts with ABPs that vary according to the subcellular compartment and function. We used a variety of approaches (including bioinformatics analyses and protein structure modeling) to determine the major differences between actin in *E. histolytica* and human monomeric actins. In transcriptomic experiments, we determined the level of actin gene expression during pathogenesis. A proteomics analysis of the actin-rich cytoskeleton revealed a number of important ABPs. Furthermore, we investigated the origin, composition, and fate of actin-rich structures in *E. histolytica* (such as “dot-like” structures, adhesion plates, stress fibers and macropinosomes) and imaged their dynamics in living trophozoites. Our experiments provided new insights into (i) the 3D structural major divergences of EhActin compared to HsActin; (ii) the genesis of actin-enriched structures in *E. histolytica*, and (iii) highlighted an important role for the Arp2/3 actin-nucleation complex in the dynamics of the actin-rich cytoskeleton.

## Materials and methods

### Cell strain and culture

The *E. histolytica* strain HM1:IMSS was cultured in TYI-S-33 medium at 37°C (Diamond et al., 1978). Drug treatments included latrunculin B (100 nM, for 15 min; this compound binds to actin monomers and prevents actin polymerization), jasplakinolide (Sigma-Aldrich, USA, at 10 μM, for 30 min; this is a filament polymerizing and stabilizing agent), and 2-fluoro-N-[2-(2-methyl-1H-indol-3-yl)ethyl]-benzamide (CK-666, Sigma-Aldrich, USA at 40 μM for 2 h; this compound binds to the Arp2/3 complex, stabilizes it in an inactive state, and prevents the formation of the active conformation). The bacterium *Escherichia coli* (One Shot TOP10, Thermo Fisher Scientific, USA) was grown in Luria-Bertani medium supplemented with ampicillin (100 μg/ml) and used for plasmids amplification.

### Expression of actin-encoding genes in *E. histolytica*

The full-length nucleotide sequences of the eight copies of the actin gene (EHI_182900, EHI_159150, EHI_142730, EHI_126190, EHI_140120, EHI_107290, EHI_163750, and EHI_043640) were retrieved from AmoebaDB (the amoeba genomics resource at http://amoebadb.org/amoeba/). The DNA sequences corresponding to the open reading frame or the 5' end of the actin gene (150 bp upstream of the ATG initiation codon) were aligned using Clustal Omega (https://www.ebi.ac.uk/Tools/msa/clustalo/). Values of actin gene expression were obtained from RNA-Seq data of *E. histolytica* in culture and interacting with human colon during intestinal infection (Weber et al., 2016).

### Structure modeling of actin proteins

The three-dimensional structure of EhActin (Uniprot P11426, 376 residues) or HsActin (Uniprot P68032, 377 residues) was predicted by homology modeling with the I-TASSER package (https://zhanglab.ccmb.med.umich.edu/I-TASSER/). The three-dimensional (3D) models were validated with Procheck, Rampage, and Verify_3D software tools (http://services.mbi.ucla.edu/Verify_3D/), and visualized using Visual Molecular Dynamics (VMD) software.

### Molecular dynamics simulations

We used the CHARMM22 force field and the TIP3P water model from the GROMACS software package (Foloppe and MacKerell, 2000). Proteins were solvated in a cubic box with 1 nm edges; 27,654 water molecules and 13 sodium ions were added for HsActin, while 30,878 water molecules and 14 sodium ions were added for EhActin. Molecular dynamics (MD) simulations were conducted with periodic boundary conditions in an isobaric-isothermal ensemble, at 300 K and 0.1 MPa for 150,000 ps. These parameters are typically used to mimic experimental conditions. The coordinates and energy data were stored every 1 ps. The atomic characteristics of HsActin and EhActin proteins were compared using the analysis tools included in the GROMACS software. The root mean square deviation (RMSD) and root mean square fluctuation (RMSF) of the backbone were calculated. The change over time in the secondary structures of both proteins was followed using the do_dssp tool in GROMACS. For these analyses, the time at which the RMSD converged was considered to be the initial step (40,000 ps for HsActin, and 10,000 ps for EhActin) for the production of simulations.

### Immunofluorescence assays

Trophozoites (4.5 × 10^4^) on slides were incubated under anaerobic conditions overnight at 37°C in TYI-S-33 medium using an Genbag Anaer (catalog number 45,534, Biomérieux, France). The medium was removed, and the cells were fixed with 37°C-prewarmed buffer containing 10 mM PIPES pH 7.4, 3 mM MgCl_2_, 1 mM EGTA pH 8, 1 mM DTT and 4% PFA (for 30 min at room temperature (RT). The fixed trophozoites were permeabilized with 0.05% Triton X-100 in PBS for 1 min. Next, the slides were washed with PBS and quenched with 50 mM NH_4_Cl for 15 min. After blocking with 2% BSA for 1 h, the slides were incubated with primary antibody for 2 h at RT, washed with PBS, and incubated with secondary antibody or phalloidin for 1 h at RT. The primary antibodies included a mouse monoclonal anti-actin antibody (clone C4, Merck Millipore, Germany, 1:200 dilution) and a rabbit polyclonal anti-Arp3 antibody (1:200 dilution, generated in the present study - see below). The secondary antibodies were goat anti-rabbit Alexa Fluor-488 or Alexa Fluor-546 antibodies, and goat anti-mouse Alexa Fluor-546 or AlexaFluor-488 antibodies (Molecular Probes, 1:200 dilution). To decorate microfilaments, we used phalloidin Alexa Fluor-488 or phalloidin Alexa Fluor-546 (Sigma-Aldrich, USA, 1:200 dilution). Lastly, coverslips were washed with BSA-free PBS, mounted with ProLong antifading reagent containing DAPI (Molecular Probes, USA), and observed under a confocal microscope (LSM700, Zeiss, Germany). Confocal planes were acquired in Z-stacks (step size: 0.5 μm) and structures were quantified in randomly selected cells from 10 fields, using a 63X objective, NA = 1.4.

The anti-Arp3 antibody was raised in rabbits (Eurogentec, Belgium) by immunization with the purified peptides 252-FKKHQAIDPISKKP and 334-LQRDURRFTDFRJQK from the amino acid sequence of *E. histolytica* Arp3 protein (locus tag: EHI_198930 and XP_647871).

### Image analysis and quantification

All image analyses were conducted using open-source Icy software (http://icy.bioimageanalysis.org; de Chaumont et al., 2012). The “Ruler Helper” plug-in was used to measure the size of structures. Colocalization of actin with the various cell markers was quantified using the “Colocalization Studio” plug-in and calculating Pearson's correlation coefficient with *p* < 0.05. The strength of association is considered as small (*PS* = 0.1 to 0.3); medium (*PS* = 0.3–0.5) or large (*PS* = 0.5–1.0). Specific regions of interest (ROIs) within the cells (i.e., adhesion plates and macropinosomes) were taken from raw image data for 54 randomly selected trophozoites. A single, representative cell from this subset is shown the results presented below.

### Construction of a recombinant actin-HaloTag plasmid

The nucleotide sequence of the *E. histolytica* HM1:IMSS strain's actin gene (EHI_163750, 1,113 bp) with *BglII* restriction sites at the 5′ and 3′ ends was synthetized by Eurofins (Brussels, Belgium). The stop codon was silenced by replacing the TAA codon by an arginine AGA codon within the *BglII* endonuclease site. Furthermore, the internal *BglII* restriction site was eliminated by the silent G255A mutation, which does not affect the protein's amino acid sequence. The gene was cloned into a pEX-K4 vector and propagated in *E. coli*. The purified recombinant plasmid was digested with *BglII* restriction endonuclease, and the resulting fragment was ligated into the *BglII* restriction site of the pHalo-Tag vector (kindly provided by Professor Tomoyoshi Nozaki) so that the HaloTag (Promega, USA) would be fused to the C-terminus of the actin protein. The construct's orientation and sequence were checked by endonuclease digestion and DNA nucleotide sequencing. The recombinant pEhEx-ActinHaloTag plasmid and the empty pHalo-Tag vector were transfected into trophozoites, as described below.

### Transfection assays

*Entamoeba histolytica* trophozoites were transfected according to a modified version of a previously published protocol (Penuliar et al., 2012). Briefly, 2 × 10^5^ trophozoites in TYI-S-33 medium were seeded on a six-well plate, and incubated under anaerobic conditions using a Genbag Anaer (catalog number 45534, Biomerieux, France) at 37°C until the cells reached 80% confluence (usually less than 24 h). Transfection was performed in OPTI-MEM media (Thermo Fisher Scientific, USA) containing L-cysteine (5 mg/ml) and ascorbic acid (1 mg/ml) adjusted to pH 6.8 (referred to as transfection medium, TM). Actin-HaloTag or HaloTag plasmid DNA (4 μg) diluted in TM (final volume: 30 μl) was mixed with 15 μl of Lipofectamine 3000 (Thermo Fisher Scientific, USA) and incubated at RT for 15 min. Next, 960 μl of TM were added to the mixture. After removing the TYI-S-33 medium, the DNA/TM/Lipofectamine mixture was added to the trophozoites, and the plate was incubated at 37°C for 3 h under anaerobic conditions. The mixtures were then transferred into a 14 ml glass tube containing TYI-S-33 medium. After overnight incubation, the transfection mixture was eliminated, fresh TYI-S-33 medium was added, and the cultures were incubated at 37°C for 24 h. Lastly, increasing amounts (2, 5, and 10 μg) of G418 (Sigma-Aldrich, USA) were added for the drug selection of transfected cells.

### Protein detection by immunoblotting

Total protein extracts were obtained from 10^6^ amoebae incubated in lysis buffer (10% SDS, 10 mM Tris/HCl) containing a protease inhibitor cocktail (20 mM leupeptine (Sigma-Aldrich, USA), 50 mM *N*-ethylmaleimide (Sigma-Aldrich, USA), 5 mM p-chloromercuribenzoate (Sigma-Aldrich, USA), 2 mM 4-(2-aminoethyl) benzenesulfonyl fluoride (Sigma-Aldrich, USA), 2 × complete mini EDTA-free (protease inhibitor cocktail, Roche, Suisse), a tablet of phosSTOP (Roche, Suisse), 10 mM E-64, 2 mM Na_3_VO_4_, 100 mM NaF, 10 mM iodoacetamide and 1% SDS). Crude cell extracts were boiled for 5 min at 100°C, cooled in ice for 1 min, aliquoted, and stored at −20°C. Protein samples (equivalent to 80,000 cells per lane) were resolved by 10% SDS-PAGE and electrotransferred onto a 0.2 μm PVDF membrane (Immobilon PSQ, Millipore). Proteins were detected by immunoblotting using mouse anti-actin monoclonal antibody (1:50 dilution; Clone C4, Merck Millipore, Germany), anti-HaloTag mouse monoclonal antibody (1:200 dilution, Promega, USA) or polyclonal anti-Arp3 (1:500 dilution, this work); the secondary antibodies were sheep peroxidase-conjugated anti-mouse (1:10,000; G&E) or anti-rabbit (1:20,000; G&E, USA) IgG. Membranes were treated with ECL Western blotting detection reagent and then exposed to Kodak Biomax film.

### Actin-HaloTag expression and cell imaging

Transfected trophozoites were grown overnight at 37°C in TYI-S-33 medium. The medium was then replaced by incomplete (serum-free) TYI-S-33 medium (TYIi) and labeled with 1 μM HaloTag tetramethylrhodamine (TMR) ligand (Promega, USA) for 15 min. After washing with TYIi, trophozoites were suspended in TYIi and seeded on 35 mm glass-bottomed imaging dishes (Ibidi, France). Images were recorded with a spinning disk confocal microscope (UltraVIEW VoX, Perkin Elmer, USA; excitation: 561 nm; objective: 63x; temperature control set to 37°C. Images were acquired with Volocity 3D image analysis software (Perkin Elmer, USA). In some experiments, 40 μM CK-666 (Baggett et al., 2012) was added for 2 h before image acquisition.

### Cell fractionation and the recovery of actin and its partners by immunochromatography

Initially, 7 × 10^6^ trophozoites were washed twice with 4°C cold PBS containing 5 mM EGTA, recovered, and lysed with 800 μL of lysis buffer (60 mM PIPES pH 7, 25 mM HEPES, 125 mM KCl, 2 mM MgCl_2_, 5 mM EGTA pH 8, 1% Triton-X-100, 0.5 mM ATP) in the presence of protease inhibitors (as described above). The mixture was centrifuged at 500 × g for 15 min at 4°C and the recovered supernatant was further centrifuged at 100,000 × g for 1 h at 4°C. Next, the pellet (i.e., the fraction not soluble in Triton-X100) was suspended in 800 μL of Tris-glycerol buffer (125 mM Tris and 20% glycerol without SDS and β-mercaptoethanol). For the immunochromatography assay, 40 μL of iron beads coupled to proteins A and G (PAG-beads, Ademtech, France) were placed in a tube on a magnetic unit until a pellet had been formed. The pellet was washed twice with 0.65% Tween 20 in PBS pH 7.5. Next, 7 μg of anti-actin C4 antibody were added for 60 min, at 4°C with agitation (1,000 rpm). Anti-GST (an irrelevant antibody) was used as a control. The tube was placed on the magnetic unit until a pellet had formed, the supernatant was removed, and the beads were recovered in 40 μl of 200 mM triethanolamine pH 9.0. The tube was again placed on the magnetic unit until a pellet had formed. The supernatant was removed, and the pellet of beads/antibody were suspended in 200 μl of 20 mM dimethyl pimelimidate dihydrochloride dissolved in 200 mM triethanolamine pH 9.0, and incubated for 60 min with agitation (1,000 rpm) at 4°C. The tube was placed on the magnetic unit, and the supernatant was removed. The reaction was stopped by adding 40 μL of 50 mM Tris pH 7.5, and the mixture was incubated for 30 min with agitation (1,000 rpm) at 4°C. The beads/antibody were washed twice and suspended in 40 μl of 50 mM glycine, 0.65% Tween 20, pH 2.7. The tube was placed on the magnetic unit. The beads were recovered in the Tris-glycerol buffer previously described, and then mixed with the above-mentioned Triton X-100 insoluble fraction overnight at 4°C with agitation (1,000 rpm). The tube was placed on the magnet unit, and the supernatant was recovered as a flow-through sample. The beads/antibody/actin were washed three times, and the supernatant was recovered as wash samples. Fifteen microliters of PAG elution buffer were added to the beads and mixed for 2 min. The supernatant containing actin and its partners was recovered as the elution sample. Lastly, 10 μl of 50 mM Tris pH 7.5 was added to neutralize the acidic samples.

### Protein analysis by liquid chromatography coupled to tandem mass spectrometry (LC-MS/MS)

The elution samples were loaded on a 12% acrylamide gel with a 4% stacking gel and electrophoresis was run until the samples had reached the separating gel. The proteins were then excised and processed for identification with LC-MS/MS, using a standard protocol (Shevchenko et al., 2006; Perdomo et al., 2016) and an Orbitrap Velos instrument (Thermo Fisher Scientific, USA) connected to a nanoUltimate 3000 HPLC system (Dionex). Mass spectrometry peak lists were generated from the raw data files using Proteome Discoverer version 1.2 (Thermo Fisher Scientific, USA). The resulting peak lists were searched with Mascot v.2.2 (Matrix science, London, UK) against the *E. histolytica* HM1:IMSS protein database from Uniprot (http://www.uniprot.org/) concatenated with known contaminants and reversed sequences of all entries. Peptide identifications were accepted if the probability in the Peptide Prophet algorithm was greater than 95.0% (Keller et al., 2002). The list of proteins was visualized and retrieved using Scaffold software (http://www.proteomesoftware.com/products/scaffold/). The various categories of identified proteins were obtained by searching with PANTHER tools (http://pantherdb.org) and InterProScan (https://www.ebi.ac.uk/interpro). Venn diagrams were generated using Venny software (http://bioinfogp.cnb.csic.es/tools/venny/index.html). The reference *E. histolytica* genome was retrieved from the AmoebaDB (http://amoebadb.org/amoeba/).

## Results

### Actin-encoding genes in *E. histolytica*

We previously identified eight copies of the actin coding gene in *E. histolytica* (EHI_182900, EHI_159150, EHI_142730, EHI_126190, EHI_140120, EHI_107290, EHI_163750, and EHI_043640), and determined that the predicted protein is phylogenetically related to amoebazoan (e.g., *Dictyostelium discoideum*) and parabasalid actins (e.g., *Trichomonas vaginalis*) (Hon et al., 2010). To study these genes, we first retrieved the corresponding nucleotide sequences from AmoebaDB and aligned them using Clustal. The gene EHI_043640 appeared as a truncated version, and so we discarded it from further analyses. The seven full-length nucleotide sequences gave a high alignment score—indicating that they are extremely conserved—and showed 93–99.7% homology (Supplemental Datasheet 1) with a total of only 17 nucleotide mismatches. The seven genes encode a protein that is 100% homol. Moreover, alignment of the 150 bp region at the 5' end of each gene also highlighted major sequence similarities between the seven full-length genes. This was particularly true for the nucleotides near the transcription initiation site, which suggests the coordinated regulation of actin gene expression (Supplemental Datasheet 1). In previous work, it was concluded that the actin gene's 5' untranslated region (UTR) contains a cAMP-response element (CRE, with the palindromic sequence TGACGTCA) and a serum-response element (SRE) box (with the CC(A/T)nGG motif) (Ortiz et al., 2000). Both motifs are involved in actin gene transcription in various eukaryotic cells. Our present analysis demonstrated that only the EHI_182900 locus carries the indicated motifs. Overall, these findings indicate that *E. histolytica'*s actin genes may correspond to recent duplications of an ancestral gene within the amoeba genome.

**Figure 1:**
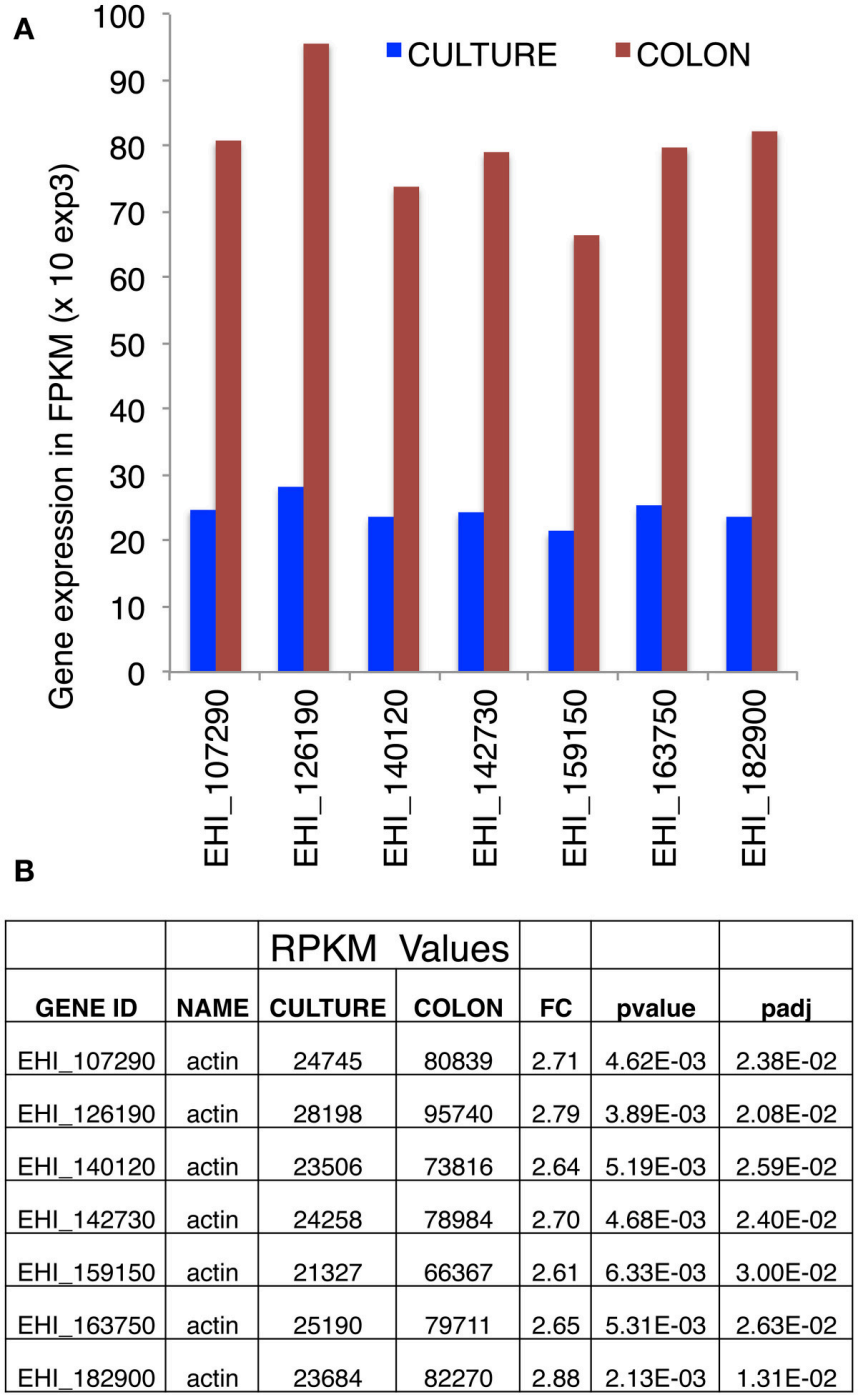
Transcriptional analysis of actin genes, obtained by RNA-Seq. **(A)** Expression of actin-encoding genes, based on RNA sequencing of *E. histolytica* transcripts in cultured trophozoites (blue bars) or after seeding onto a human colon explant (red bars) by Weber et al. (2016) (the accession numbers are taken from AmoebaDB). **(B)** Counted reads. In cultured trophozoites, each of the seven actin-encoding genes gave similar numbers of reads. This was also true for trophozoites seeded onto a human colon explant. There was a 2.5-fold overall increase in actin transcription during pathogenesis. The numbers of reads are quoted with the respective probability values and adjusted probability values. FPKM: fragments (reads) per kilobase of transcript per million fragments mapped.

To investigate actin gene expression, we took advantage of our literature transcriptome data concerning *E. histolytica* in culture or during parasite interaction with the human intestinal colon (Weber et al., 2016). Although the nucleotide sequence (reads) in the amoeba genome were mapped stringently as single reads (meaning that a read is allowed to map only to one gene), the high level of nucleotide sequence conservation in actin genes prevented us from determining the specific features of transcription for each individual gene. However, taking these data as a whole, we found that the seven full-length copies of actin gene are expressed in parasites cultured *in vitro* and during infection of the human intestine. A marked (2.5-fold) overall upregulation of gene expression was observed during tissue invasion (Figure 1).

### Dynamic structure of monomeric EhActin vs. monomeric HsActin

**Figure 2:**
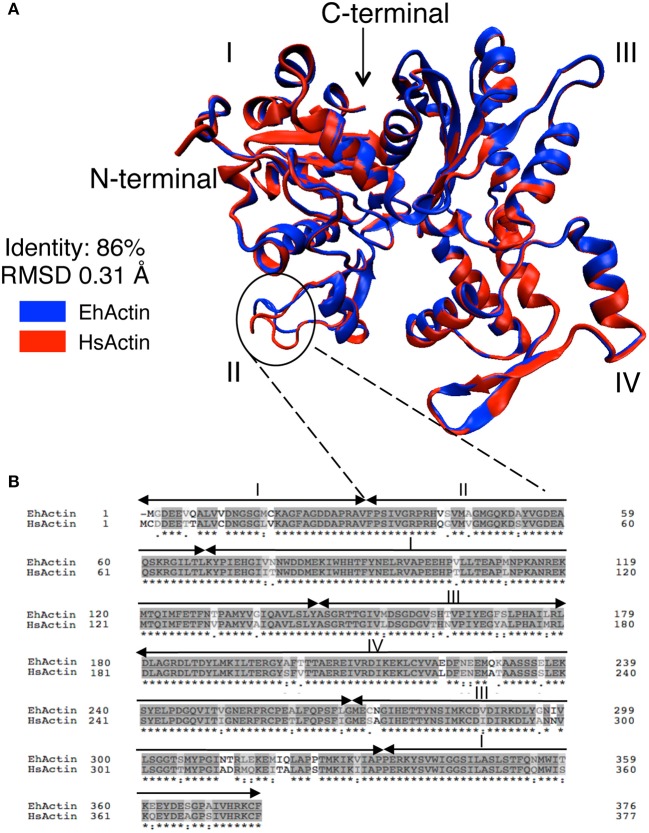
Comparison of 2D amino acid sequences and predicted 3D structures of EhActin vs. HsActin. **(A)** The 3D structure of EhActin (in blue, Uniprot P11426) was obtained with the I-TASSER package using the 3D structure of HsActin (in red, Uniprot P68032) as the template. Amino acid alignment starts at amino acid D3 in EhActin. The typical subdomains I, II, III, and IV are indicated. **(B)** Comparison of amino acid sequences of both actins after alignment using BLASTp. Note the major differences at the amino-terminus.

To gain insight into the structural differences between EhActin and HsActin, we first conducted *in silico* molecular modeling experiments. Given that the full-length amino-acid sequences of *E. histolytica* and human proteins share 86% identity, this modeling was relatively straightforward. Overall, the two proteins showed a highly conserved folding pattern, with an RMSF of 0.31 Å. As in the human protein, amoebic actin has 17 beta strands and 15 alpha helices, which form the four typical domains seen in actins. However, there are differences between the respective 3D structures—mainly in the subdomain II (Figure 2A), as a result of the substitution of non-polar residues in HsActin by polar residues in EhActin (V42/Q43; A46/S47) and, conversely, the substitution of polar residues by non-polar residues (S43/G44) (Figure 2B). Interestingly, Q43 and G44 in amoebic actin correspond to Q40 and G41 in HsActin, which are involved in the DNAse I interaction (Wriggers and Schulten, 1997). In addition, G41 is involved in HsActin interaction with Thymosin ß4, an actin sequestering protein that prevents G-actin association to microfilaments (Domanski et al., 2004).

**Figure 3:**
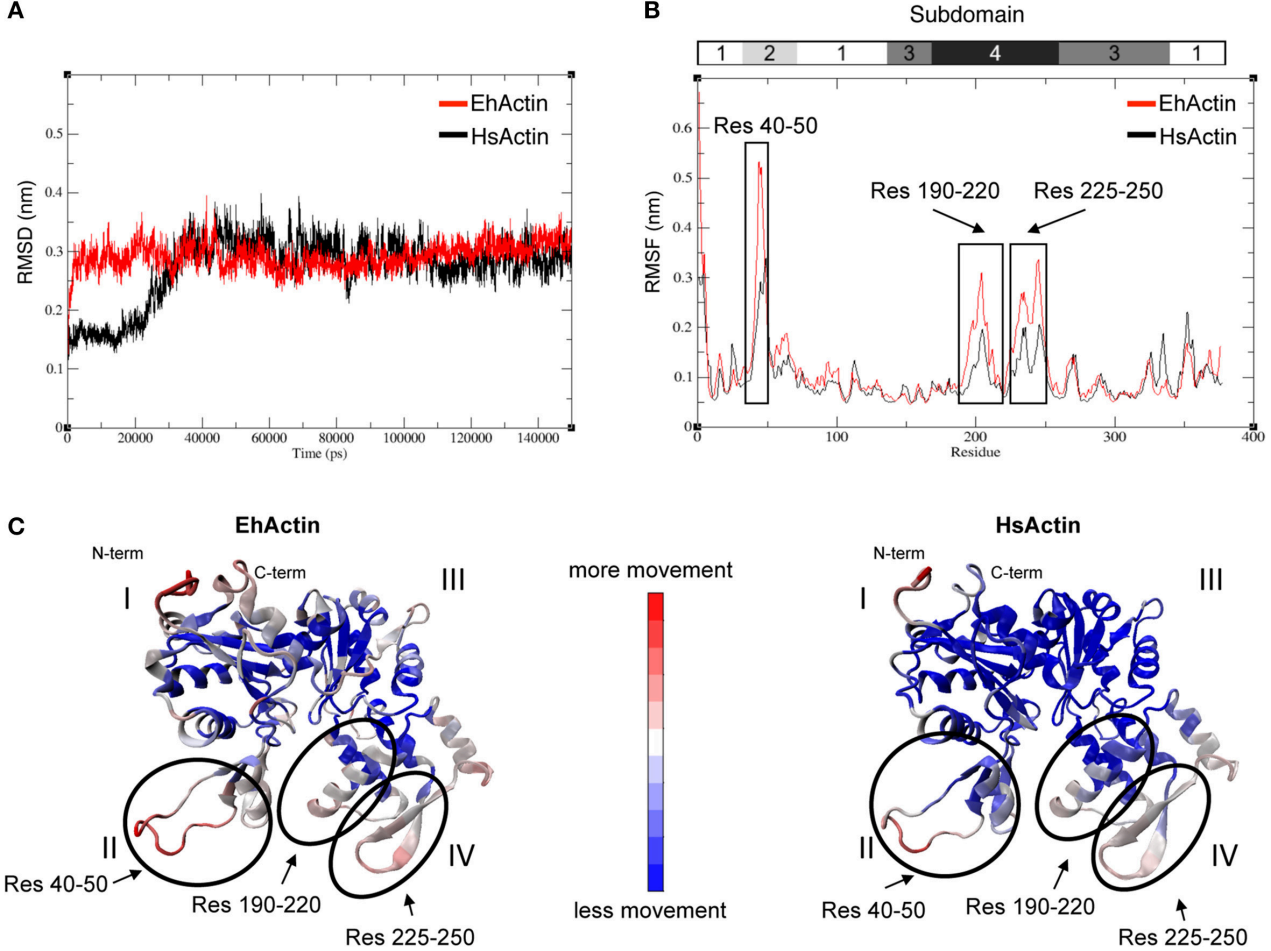
Molecular dynamics (MD) simulation of EhActin and HsActin. Backbone RMSD **(A)** and RMSF **(B)** values of EhActin (in red) and HsActin (in black). **(C)** Representation of average 3D structures obtained from MD simulations of EhActin (left) and HsActin (right). The colored scale bar indicates the intensity of movements within protein 3D domains; these movements are presented in Supplemental Videos 1, 2.

To establish whether these amino acid sequence differences have an effect on protein behavior, we performed MD simulations. We notably described the proteins' flexibility and compared structural parameters obtained from the dynamic trajectories. Amoebic actin showed a rapid increase in the RMSD during the first 5,000 ps, whereas this production step required about 30,000 ps for HsActin. The proteins then reached equilibrium and oscillated within an interval of 0.25–0.35 nm; there were no significant interval differences between the two (Figure 3A) (Supplemental Videos 1, 2). The respective RMSF profiles revealed that subdomains II and IV are the most mobile parts in both proteins, followed by subdomain I (Figure 3B). The same conclusion can be drawn from observation of the average 3D structure in MD simulations (Figure 3C). Importantly, a detailed analysis of RMSD values showed that the region spanning residues 40–50 (corresponding to subdomain II) is more mobile in EhActin than in HsActin. The next most mobile region includes residues 190–220 and 225–250 of subdomain IV (Figure 3B). Overall, the 3D structural dynamics data indicate that subdomain II is the protein region with the greatest divergence between the two actins.

### Actin and microfilaments form diverse actin-rich structures

**Figure 4:**
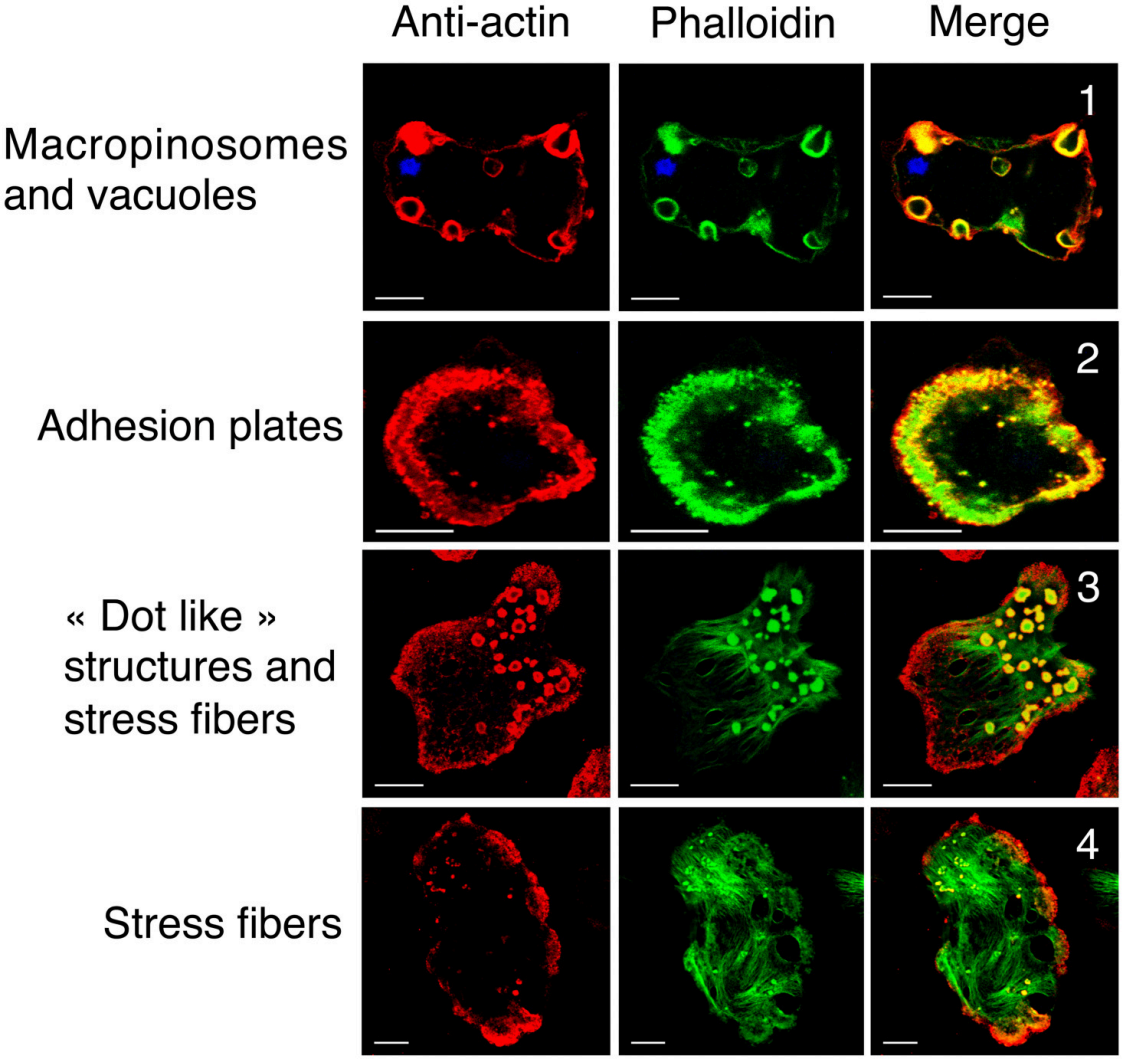
Cellular localization of G-actin and F-actin in wild-type *E. histolytica* trophozoites. Actin and microfilaments form diverse actin-rich structures running from the bottom to the top of representative trophozoites. Confocal microscopy images in various focal planes show G-actin (in red, detected by anti-actin antibody) and F-actin (in green) detected by fluorescent phalloidin. Scale bar: 10 μm. Both G-actin and F-actin are present in actin-rich structures, including (1) macropinosomes, the cortical cytoskeleton, vacuoles, (2) adhesion plates and (3) “dot-like” structures. (4) Most of the actin in stress fibers is present as F-actin. The Pearson coefficients for correlations between the structures observed throughout the Z-stack were 0.67, 0.39, 0.5, 0.16 for macropinosomes, adhesive plates, dot-like structures, and stress fibers, respectively. These values indicate a significant level of colocalization with the highest level for macropinosomes.

**Figure 5:**
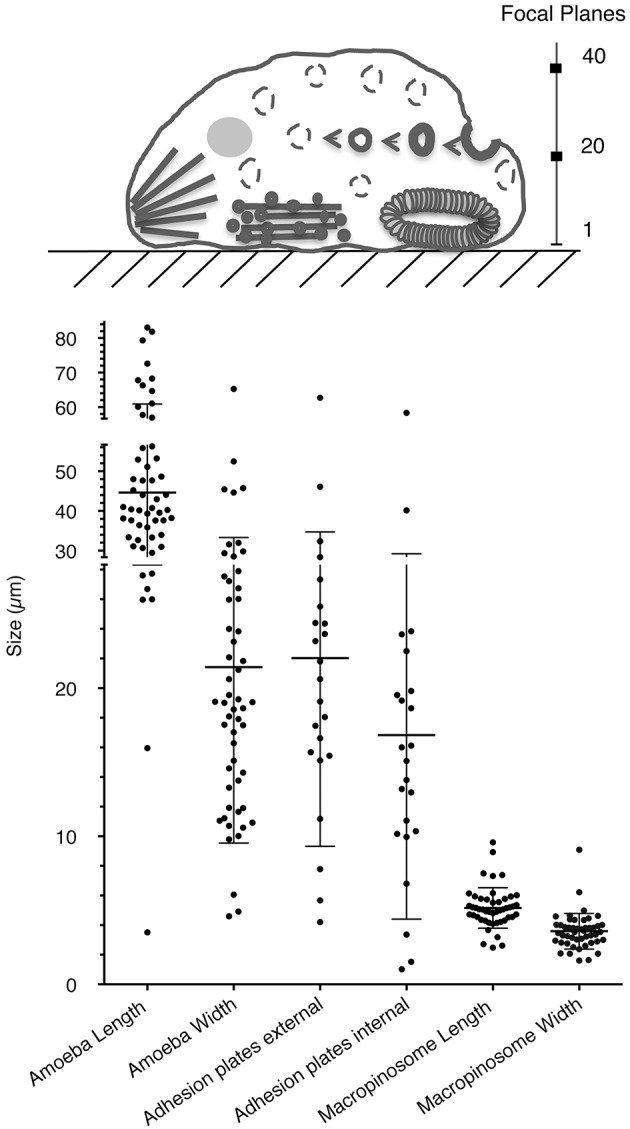
Quantification of actin-rich structures within *E. histolytica*. **(A)** Schematic representation of a trophozoite, highlighting the two cellular ROIs used to scan the various focal planes and thus describe the actin-rich structures. Macropinosomes and vacuoles are found in all planes but are mainly outside adhesive structures. Adhesion plates and “dot-like” structures are found at the bottom of the cell, from frames 1 to 10. Stress fibers can be observed at the bottom of the cell (from planes 1 to 5), and they notably accumulate at the rear of the polarized cell. **(B)** The number and size distributions of actin-rich plates and macropinosomes in 54 randomly selected trophozoites (length: 45 μm; width: 22 μm). Note the presence of large adhesion plates (in 23 of the 54 cells; external diameter: 22 μm; internal diameter: 16 μm) and macropinosomes (in 51 of the 54 cells; length: 5 μm; width: 3 μm. Both structures contain concentrated actin, as revealed by staining with both an anti-actin antibody and phalloidin.

The presence of a single actin protein in *E. histolytica* and the marked 2D and 3D structural differences with respect to HsActin prompted us to investigate the dynamics of the actin-rich structures in this parasite. In particular, the stress fibers that are preponderant in eukaryotic cells are less visible in the highly mobile amoeba. To determine the distribution of actin and the various actin-rich structures in *E. histolytica* trophozoites, we used confocal microscopy and immunodetection with the C4 monoclonal anti-actin antibody that binds to all forms of actin in eukaryotic cells (including unicellular organisms like *E. histolytica*) (Lessard, 1988). We also used phalloidin to detect F-actin-containing microfilaments. In each scanned cell (approximately 45 μm long and 22 μm wide), we identified and counted actin-rich structures over a distance of 10 μm (20 focal planes of 0.5 μm; Figure 4). Two ROIs were defined in each trophozoite, i.e., a lower part (focal planes 1–10) and an upper part (focal planes 11–20). In both ROIs, the cortical cytoskeleton was clearly seen around the cell membrane. The lower part of the cell contained stress fibers, dot-like contact points, and large adhesion plates, whereas the upper part of the cell contained small cytoplasmic dots and multiform vesicular structures. The latter included large, endocytic vacuoles reminiscent of the macropinosomes through which cells internalize fluid. We then quantified the number and size of actin-rich adhesive and macropinosome-like structures in 54 trophozoites by analyzing the confocal microscopy images after fluorescence staining (Figure 5). We found that 23 of the 54 *E. histolytica* trophozoites displayed large adhesion plates (with an external diameter of up to 22 μm and an internal diameter of up to 16 μm) containing actin stained by both the anti-actin antibody and phalloidin. Furthermore, 51 of the 54 trophozoites contained macropinosomes, which measured (on average) 5 μm in length and 3 μm in width. When we used latrunculin B and jasplakinolide to inhibit microfilament dynamics, the actin-rich structures disappeared; most of the actin aggregated inside the cells (Supplemental Figure 1), and the trophozoites became more spherical.

### Dynamics of actin-rich structures in living *E. histolytica*

**Figure 6:**
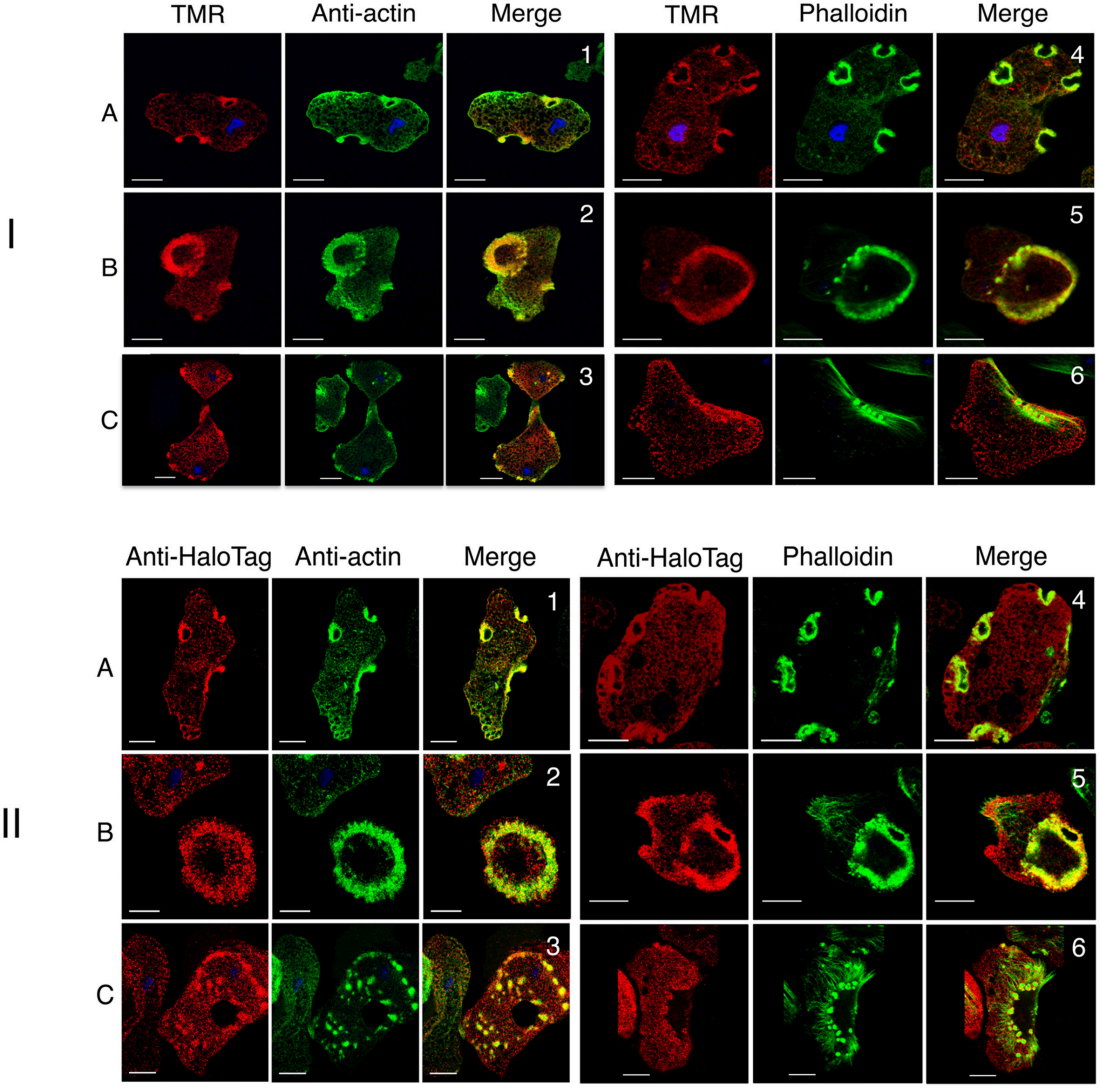
Cellular analysis of actin-HaloTag in *E. histolytica* transfectants. Actin-HaloTag-transfected trophozoites were fixed and analyzed using confocal microscopy. **(I)** The TMR probe labeled the actin-HaloTag fusion protein throughout the cell and colocalizes with actin. Micrographs correspond to focal planes at the top (A), or at the bottom (B,C) of the cells, showing actin-HaloTag (in red) detected by TMR, and G-actin and F-actin (in green) detected using an anti-actin antibody or fluorescent phalloidin, respectively. Scale bars: 10 μm. Actin-HaloTag was present in actin-rich structures, (1 and 4) macropinosomes, (2 and 5) adhesive plates, (3 and 6) “dot-like” structures, and stress fibers. Colocalization (Pearson's correlation coefficient: 0.63, 0.5, 0.28, 0.44, 0.62, and 0.15 respectively) was assessed for actin-rich structures. The values indicate a high level of colocalization, with the lowest level observed for stress fibers. **(II)** The anti-HaloTag antibody labels the actin-HaloTag fusion protein throughout the cell and colocalizes with actin. Micrographs correspond to focal planes at the top (A), or at the bottom (B,C) of the cells, and show actin-HaloTag (in red) detected by TMR, and G-actin and F-actin (in green) detected using an anti-actin antibody or fluorescent phalloidin, respectively. Scale bar: 10 μm. Both types of actin colocalized in actin-rich structures including macropinosomes (1 and 4), large adhesion plates (2 and 5), stress fibers, and “dot-like” structures (4 and 6). F-actin exhibited low anti-HaloTag staining. Colocalization (Pearson's correlation coefficient: 0.67, 0.51, 0.47, 0.48, 0.38, and 0.32, respectively) within the structure was assessed. These values indicate a significant level of colocalization, with the lowest level observed for stress fibers.

To examine the dynamics of actin-rich structures in *E. histolytica*, we transfected trophozoites with the actin-HaloTag construct as described in the Material and Methods section. DNA plasmid sequencing confirmed the quality of the construct, in which HaloTag is fused to the carboxy-terminal end of actin. We used confocal microscopy to check that the actin-HaloTag fusion protein was present in trophozoites and that it colocalized with actin (Figure 6). As expected, actin-HaloTag was bound by both the anti-HaloTag antibody and HaloTag's TMR fluorescent ligand. Structures such as macropinosomes, “dot-like” structures, stress fibers and large adhesion plates contained the actin-HaloTag fusion protein and F-actin (as identified by staining with phalloidin or anti-actin antibody) (Figure 6). Transfected and non-transfected cells did not appear to differ with regard to the abundance of actin-rich structures. The presence of the actin-HaloTag fusion protein was further examined by immunoblotting crude protein extracts from transfected trophozoites (Supplementary Figure 2A). Although the fusion protein was recognized by the anti-HaloTag antibody and had the right molecular mass, we did not find a protein signal when the anti-actin antibody was used in the immunoblot—suggesting that when extracted from cells, the fusion protein may have a lower affinity for the C4 antibody.

**Figure 7:**
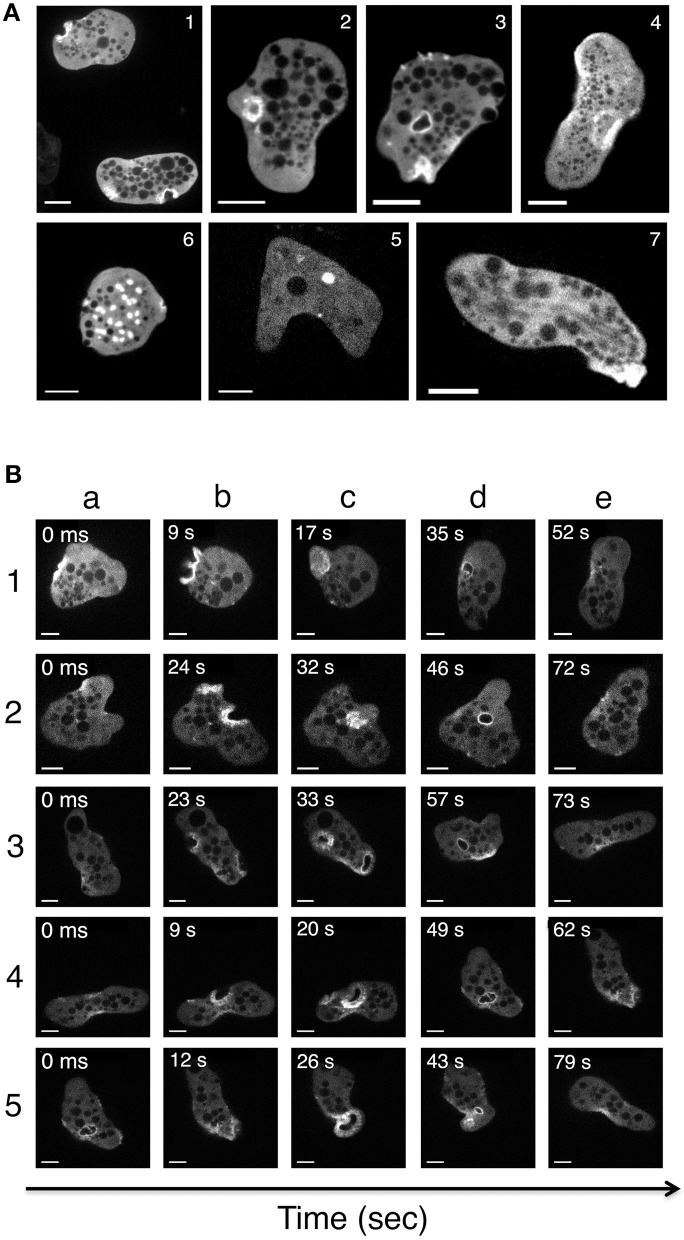
Imaging of actin-rich structures in living *E. histolytica* cells. Trophozoites expressing actin-HaloTag were viewed with laser spinning-disk video microscopy. Over time, several structures were visualized (Supplemental Video 3). Independent micrographs were selected from the videos and are shown here to illustrate the actin-rich structures present in the actin-HaloTag-transfected trophozoites. **(A)** Micrographs were taken from various amoeba. (1) Stroma-like invaginations (diameter: 5 μm). (2) Early-stage macropinosomes (diameter: 5 μm). (3) A late-stage macropinosome and a new invagination (diameter: 5 μm). (4) Adhesion plates (large structures; diameter: 10 μm). (5) An intracellular vacuole (diameter: 2 μm). (6) “Dot-like” structures; diameter: 1 to 3 μm). (7) Actin filaments at the rear of a polarized cell. Scale bar: 10 μm. **(B)** Micrographs of a single motile trophozoite (Supplemental Video 3) in which all the actin-containing structures appear one after the other. Five different events were selected as examples. Note (i) the initial accumulation of actin at the cortical cytoskeleton (1a, 2a, 4a), (ii) cell surface deformation and actin ring formation (all b lines), (iii) the closure of the macropinosome (1c, 3c, 4c), (iv) the migration of an actin-rich macropinosome to the internal cell compartments (2d, 3d, 4d) and (v) the detachment of actin from the macropinosome. In event 5, a large adhesive plate forms at the rear of the cell (5c). Scale bar: 10 μm.

We next imaged actin-rich structures in live cells (transfected with actin-HaloTag or HaloTag) using video microscopy and a spinning disk laser microscope. Remarkably, the various actin-rich structures changed very rapidly over time. Figure 7A shows a series of micrographs from various amoebae, highlighting the polymorphism of the actin-rich structures. Figure 7B depicts the course of events in a single amoeba (Supplemental Video 3). Actin-HaloTag was present, and gave a strong fluorescent signal in cell-adhesive structures (dots and plates), vacuoles, macropinosomes, and stress fibers. These fibers were present at the rear of the polarized cells and near to the plasma membrane. No signals were observed in control cells (e.g., wild-type cells or Halo-Tag-treated cells). Actin-HaloTag was recruited to the macropinocytic cup in less than 9 s. Next, the internalized macropinosomes that formed after 20–30 s were entirely covered by actin, and moved through the cytoplasm until actin disappeared from the vesicle. In some cases, the vesicle appeared to fuse with other resident endomembrane compartments. Overall, the macropinosomes' turnover time was roughly 50–60 s—indicating that actin-rich cytoskeleton is rapidly reorganized within these structures.

### Actin and ABPs in the *E. histolytica* cytoskeleton

To gain insight into the ABPs associating with the amoebic cytoskeleton, we purified actin and its partners by immunoaffinity chromatography of the cytoskeleton-enriched protein fraction. The recovered proteins were identified using LC-MS/MS. Three independent experiments were performed, and only proteins with at least two peptides were considered in the subsequent bioinformatics analysis. Using a Venn diagram, we determined that 266 proteins were present in two or three independent experiments. The PANTHER tools and manual annotations enabled us to identify cytoskeleton-related proteins, small GTPases involved in cytoskeleton regulation, and proteins related to membrane traffic. The protein families and classes are listed in Supplemental Table 1. By applying stringent criteria, we focused on 14 proteins that bound to the actin-rich cytoskeleton (Table 1). Along with actin, the most strongly represented family was the Arp2/3 complex involved in actin nucleation and which contains seven actin-related proteins: Arp2 (44 kDa), Arp3 (47 kDa), ARPC1 (40 kDa), ARPC2 (35 kDa), ARPC3 (21 kDa), ARPC4 (20 kDa), and ARPC5 (16 kDa). All the subunits other than ARPC3 and ARPC5 were found in the proteomic analysis. Arp2 and Arp3 fold into a structure that is similar to that of actin, and act as monomer nucleators. We identified a number of other proteins: the heavy chain of myosin II, which binds F-actin and is responsible for cell contraction and motility (Arhets et al., 1998); filopodin, which contains a FERM domain involved in protein membrane binding (Chishti et al., 1998) and was previously found in the uropod of moving *E. histolytica* (Marquay Markiewicz et al., 2011) and in phagosomes (Marion et al., 2005); profilin, which sequesters G-actin and thus blocks actin polymerization (Binder et al., 1995); coronin, which participates in cell migration and vesicular trafficking in eukaryotes (de Hostos et al., 1991) but has not been studied in *E. histolytica*; and cyclase-associated protein (CAP), a highly conserved protein that links nutritional response signaling to the cytoskeleton via its actin-binding carboxy-terminal end (Iwase and Ono, 2016). We also found 12 small GTPases (Supplemental Table 1), including 7 Rabs and 3 Rhos that are all expected to regulate cytoskeleton function and vesicle trafficking. Lastly, 11 proteins were associated with the endomembrane system traffic, including endocytic compartments (Supplemental Table 1).

**Table 1:** Proteins identified in the cytoskeleton enriched fraction.

**Gene ID**	**Uniprot**	**Description**	**Family/Subfamily**	**Protein class**	**Manual**
EHI_107290	B1N2P0	Actin	Actin, cytoplasmic 1-related (PTHR11937:SF155)	Actin and actin related protein(PC00039)	ACTIN
EHI_111050	C4M5I6	Actin-like protein	Actin-related protein 2(PTHR11937:SF37)	Actin and actin related protein(PC00039)	ARP2
EHI_198930	C4LWT9	Actin-related	Actin-related protein 3 (PTHR11937:SF31)	Actin and actin related protein(PC00039)	ARP3
EHI_045000	C4LTB5	Actin-related protein 2/3 subunit 1A	Actin related complex P41 subunit (PTHR10709:SF2)	Actin family cytoskeletal protein(PC00041)	ARPC1
EHI_030820	C4LSV0	Actin-related protein 2/3 subunit 4	Actin-related protein 2/3 complex subunit 4 (PTHR22629:SF0)	Actin family cytoskeletal protein(PC00041)	ARPC4
EHI_136150	C4M295	Adenylyl cyclase-associated	Adenylyl cyclase-associated protein (PTHR10652:SF0)	Actin family cytoskeletal protein(PC00041)	CAP
EHI_091250	C4M782	Arp2/3 complex 34 kDa subunit	Actin-related protein 2/3 complex subunit 2 (PTHR12058:SF0)		ARPC2
EHI_083590	C4M943	Coronin	Coronin-A (PTHR10856:SF39)		Coronin
EHI_167130	C4LYB3	Filopodin	RHEA, Isoform B (PTHR19981:SF1)		Filopodin
EHI_001070	C4M1I7	LIM zinc finger domain	Paxillin, Isoform F (PTHR24216:SF8)		
EHI_161940	C4M6N7	LIM zinc finger domain	Subfamily not named (PTHR24206:SF55)	Actin family cytoskeletal protein(PC00041)	
EHI_096420	C4LWF5	LIM zinc finger domain	LIM domain-containing protein E (PTHR24206:SF56)	Actin family cytoskeletal protein(PC00041)	
EHI_194520	C4LZW4	LIM zinc finger domain	Subfamily not named (PTHR24215:SF22)	Actin family cytoskeletal protein(PC00041)	
EHI_110180	C4LU72	Myosin heavy chain	Myosin heavy chain, muscle (PTHR45615:SF27)		Myosin II
EHI_176140	C4MB21	Profilin	Profilin-1 (PTHR11604:SF19)	Non-motor actin binding protein(PC00165)	Profilin
EHI_010530	C4LYU0	Tubulin alpha chain	Tubulin alpha-2 chain-related (PTHR11588:SF297)	Tubulin(PC00228)	α-tubulin
EHI_049920	C4LUJ0	Tubulin beta chain	Tubulin beta chain (PTHR11588:SF9)	Tubulin(PC00228)	ß-tubulin

### Arp2/3 is involved in macropinosome and adhesion plates formation

**Figure 8:**
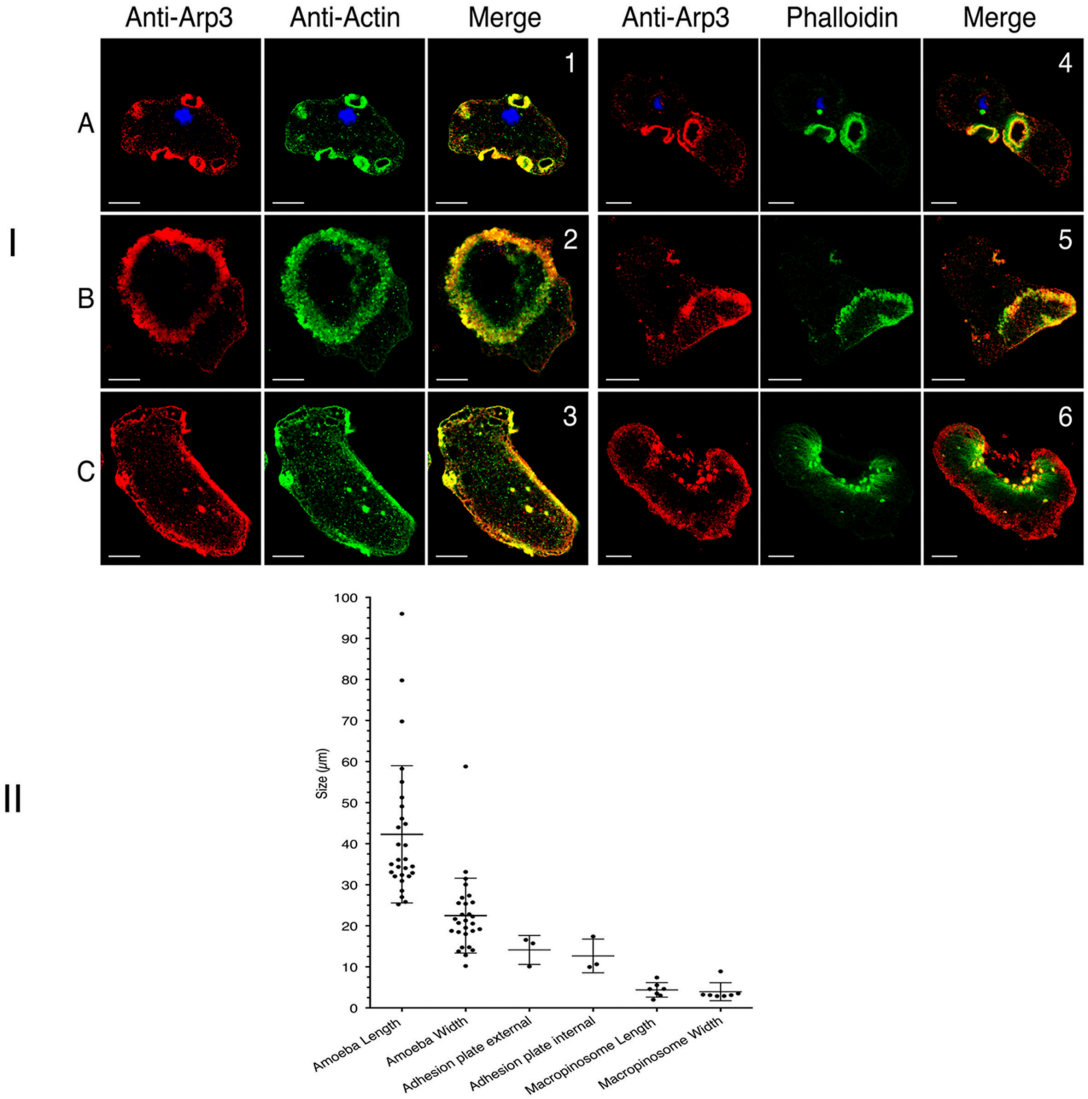
Cellular localization of Arp3 in *E. histolytica*. **(I)** Cellular localization of Arp3 (red) with G-actin (green) or F-actin (green) in wild-type *E. histolytica* trophozoites, assessed using immunofluorescence and confocal microscopy. Images were taken at the top (A) or the bottom (B,C) of the cells. Scale bar: 10 μm. Both types of actin colocalized in actin-rich structures, such as macropinosomes (1 and 4), adhesion plates (2 and 5), dots and stress fibers (3 and 6). Arp3 is present in all actin-rich structure except stress fibers (Pearson's correlation coefficient: macropinosomes (0.78 and 0.77); adhesion plates (0.53 and 0.53); dots (0.44 and 0.38) and stress fibers (0.03). These values indicate a significant level of colocalization, with the exception of long stress fibers (green stained in 6). **(II)** Inhibition of the Arp 2/3 complex reduced the number of adhesion plates and macropinosomes. The figures shows the number and size distributions for actin-rich adhesion plates and macropinosomes after the treatment of cells (wild-type *E. histolytica* or transfected trophozoites) with the Arp 2/3 complex inhibitor CK-666.

Due to the prominence of Arp2/3 within the actin-rich cytoskeleton (as analyzed by proteomics), we studied the complex's involvement in the dynamics of the above-mentioned actin-rich structures. To this end, we first prepared an anti-Arp3 antibody (see the Materials and Methods section). In immunoblots of an *E. histolytica* crude extract, the antibody bound to a single 47 kDa protein as expected (Supplemental Figure 2B). To examine the localization of Arp3 protein in trophozoites, we performed confocal microscopy and immunofluorescence experiments on wild-type cells and actin-HaloTag transfected cells. The results showed that Arp3 colocalizes with G-actin and F-actin in macropinosomes, dots, adhesion plates, and the cortical cytoskeleton but not in stress fibers (Figure 8I). CK-666 is a recently discovered small-molecule inhibitor of the Arp2/3 complex that binds at the interphase between the subunits and stabilizes an inactive conformation (Baggett et al., 2012). Treatment with CK-666 greatly reduced the number of adhesion plates and macropinosomes per cell (respectively seen in 3 and 6 of the 28 cells), whereas the number of stress fibers did not change (Figure 8II). These findings highlight the implication of Arp2/3 complex in adhesion plates and macropinosomes formation.

## Discussion

*Entamoeba histolytica'*s ability to move, divide, kill, and phagocytose human cells requires actin, which is widely distributed throughout the amoebic cytoplasm. The continuous polymerization/depolymerization of actin enables the deployment of several different structures. The *E. histolytica* genome carries seven full-length actin-encoding genes but (unlike higher organisms) expresses a single, conventional isoform. The selective evolutionary advantage of maintaining multiple copies of genes coding for the same protein has not been elucidated. Nevertheless, in view of the nucleotide sequence similarities between the actin genes, one can reasonably hypothesize that the actin gene family arose from duplications of an ancestral gene. As in the case of actin genes in *E. histolytica*, gene duplication is also seen in *E. dispar* and *E. invadens* (Hon et al., 2010). In phylogenetic terms, amoebic actin is closely related to actin from *Trichomonas vaginalis* (another infectious parasite) and the free-living amoeba *Dictyostelium discoideum*.

The actin genes have sequence similarities in the 5'UTR, which suggests the existence of common transcription factor binding sites. In previous studies, potential regulatory DNA motifs (e.g., CRE and SRE) were described in actin's 5'UTR (Ortiz et al., 2000); here, we conclude that these motifs are only present at the EHI_182900 actin locus. Nevertheless, all seven full-length loci share consensus DNA stretches at regions very close to the transcription start site. Potentially these common DNA stretches may regulate all actin genes transcription. This proximity is observed for many genes in *E. histolytica*'s very compact genome (Loftus et al., 2005). Although actin gene expression is abundant in *E. histolytica*, it is difficult to draw conclusions as to the specific contribution of each gene to the actin mRNA levels. Nevertheless, invasion of the human intestine led to a 2.5-fold overall increase in actin gene transcription. The increase in actin mRNA abundance may be justified by the potential need for more protein during infection-related processes such as cell motility and phagocytosis. Furthermore, it is possible that actin itself regulates the gene expression profile during the infectious process; indeed, monomeric nuclear actin reported regulates gene expression, modifies nuclear content, and maintains genome integrity in eukaryotic cells (Virtanen and Vartiainen, 2017).

We found that most of the structural differences between EhActin and HsActin concerned domain II of the protein. In particular, EhActin and HsActin differ with regard to the amino acids involved in the interaction with DNAse I. Subdomain II is also involved in the G-actin interaction with thymosin β4 which sequesters actin monomers, how the instability of subdomain II can influence this interaction in E. histolytica is an open question. This observation suggests that the loop between amino acids 30 to 52 in monomeric actin is a prime potential target for exploitation in drug screening. Two loops in domain IV (at amino acids 190–220 and 225–250) are also good potential targets.

Live cell imaging enabled us to establish the first image-based atlas of actin-containing structures in *E. histolytica*: actin-dots, adhesion plates, and macropinosomes. It has been reported that actin dots accumulate on the ventral side of trophozoites; this process has been linked to signal pathways that depend on extracellular matrix cell surface receptors (e.g., fibronectin (FN) receptors) regulated by the activity of the small GTPase Rab21 (Emmanuel et al., 2015). The presence of these dots in the non-FN-activated amoebae studied here suggests that they accumulate when the cell's displacement is slowed down by adhesive FN signaling. Adhesion plates have been previously observed in *E. histolytica* seeded on FN (Vázquez et al., 1995). The plates are enriched in cytoskeletal proteins such as actin and the associated myosin I, myosin II, alpha-actinin, and tropomyosin.

*Entamoeba histolytica* accumulates fluid-phase markers by macropinocytosis (Meza and Clarke, 2004); here, we observed a link between macropinocytosis and the actin-rich cytoskeleton. By taking advantage of the properties of a fusion protein between actin and HaloTag (a fluorescent tag that is well suited to use in anaerobes), we gained information on the dynamics of macropinocytosis in *E. histolytica*. The vacuoles form and circulate in as little as 50–60 s. Actin then detaches from the vesicle, which eventually fuses with the resident internal endomembrane system. Macropinocytosis differs from phagocytosis, and is conserved among amoeboid eukaryotic cells. A large body of evidence indicates that this feeding phenomenon depends on Ras signaling pathways. To form, macropinosomes start by accumulating actin beneath the plasma membrane in order to produce a ring. Upon membrane ruffling, the ring closes by membrane fusion to produce an internal vesicle filled with external fluid (for a recent review, see Bloomfield and Kay, 2016). These large structures (up to 5 μm in diameter) occur in a wide range of cell types, including immune cells (e.g., macrophages and dendritic cells) and amoebae. It was recently observed that macropinocytosis and cell motility are not compatible in dendritic cells during antigen presentation (Chabaud et al., 2015) or in *D. discoideum* during chemotaxis (Veltman et al., 2014). This is not the case in *E. histolytica*, since our video microscopy studies showed that macropinosomes formed in highly motile cells. However, we have not yet looked at whether chemotaxis—which is important for pathogenesis in *E. histolytica*—competes with macropinosome formation.

Our rigorous proteomic study highlighted the abundant presence of the Arp2/3 complex in the cytoskeleton fraction. This complex binds pre-existing microfilaments, induces an actin nucleation step, and this starts the filament branching process. The new branch forms at an angle of 70° to the supporting filament, leading to a so-called dendritic network of actin (Pollard, 2016). We found that the Arp3 subunit of the Arp2/3 complex in *E. histolytica* is associated with actin dots, adhesion plates and macropinosomes but not stress fibers. Experiments with the inhibitor CK-666 showed that the Arp 2/3 complex was essential for the formation of adhesion plates and macropinosomes. The functional study of macropinocytosis in *E. histolytica* is in its early stages. Based on the actin-HaloTag construction and the information gained from our proteomic studies, we hope to further elucidate the role of this nutrient pathway in the parasite's invasion of the intestine.

## Author contributions

MM, NH-C, JO-V, and SS performed the experiments; JO-V, LM, and NG performed the bioinformatics analyses; MM, J-CO-M, LM, and NG designed the study and wrote the manuscript.

### Conflict of interest statement

The authors declare that the research was conducted in the absence of any commercial or financial relationships that could be construed as a potential conflict of interest.

## Supplementary material

Supplemental Figure 1 *E. histolytica* actin-rich structures belong to the actin-rich cytoskeleton. Confocal image micrographs from Z-Stack show G-actin (in red, detected by anti-actin antibody) and F-actin (in green, detected by fluorescent phalloidin). Scale bar: 10 μm. **(A)** Wild-type trophozoites. **(B)** Trophozoites treated with latrunculin B or jasplakinolide. Scale bar: 10 μm. Treated amoebae displayed an aggregation of actin filaments and adopted a spherical shape, due to the inhibition of actin cytoskeleton dynamics.

Supplemental Figure 2 Expression of actin-HaloTag in *E. histolytica* trophozoites. A sample of amoebic extracts (equivalent to 80,000 cells) was resolved by SDS-PAGE and immunoblotted as described in the Material and Methods section. **(A)** Electrophoretic analysis of proteins from transfected and wild-type amoebae, for the detection of actin and actin-HaloTag. Actin (WT, 42 kDa), HaloTag (H, 34 kDa), and actin-HaloTag (AH, 76 kDa) were detected in the respective protein extracts. As expected, only endogenous actin was detected in both transfected and wild-type amoebae. The anti-HaloTag antibody detected an additional band (at roughly 30 kDa) in the actin-HaloTag protein extract; this may reflect degradation of the fusion protein. Line1 corresponds to Actin-HaloTag cells; line 2 is HaloTag cells and line3 is wild-type amoeba. **(B)** Electrophoretic analysis of proteins from wild-type amoeba for the detection of Arp3. A single band was seen at 47 kDa.

Supplemental Table 1 Proteins identified by proteomics.

Supplemental Video 1 Molecular dynamics simulation of HsActin. The video was made with VMD software and shows the behavior of human actin during 150,000 ps of simulation.

Supplemental Video 2 Molecular dynamics simulation of EhActin. This video was made with VMD software and shows the behavior of EhActin during 150,000 ps of simulation.

Supplemental Video 3 Changes over time in actin-HaloTag structures. Transfected trophozoites (actin-HaloTag) in TYIi medium at 37°C were imaged using a spinning disc confocal microscope. All images were acquired using a 63X objective in a selected confocal plane. For video reconstruction, 582 frames were taken into account and the video speed is 15 frames per second. The scale bar corresponds to 10 μm.

Supplemental Datasheet 1 Nucleotide sequence analysis of actin-encoding genes in *E. histolytica* HM1:IMSS strain. The DNA sequences were taken from AmoebaDB and aligned using CLUSTAL. The document indicate identity (stars) and homology (double dots) between the DNA sequences in coding regions **(A)** and promoter regions (150 bp upstream of the ATG initiation codon) **(B)**.
