# Supplementary figures and images for: Morphodynamics of the Actin-Rich Cytoskeleton in Entamoeba histolytica
Source: Front Cell Infect Microbiol. 2018 May 29;8:179. doi: 10.3389/fcimb.2018.00179 (PMC5986921; doi:10.3389/fcimb.2018.00179)

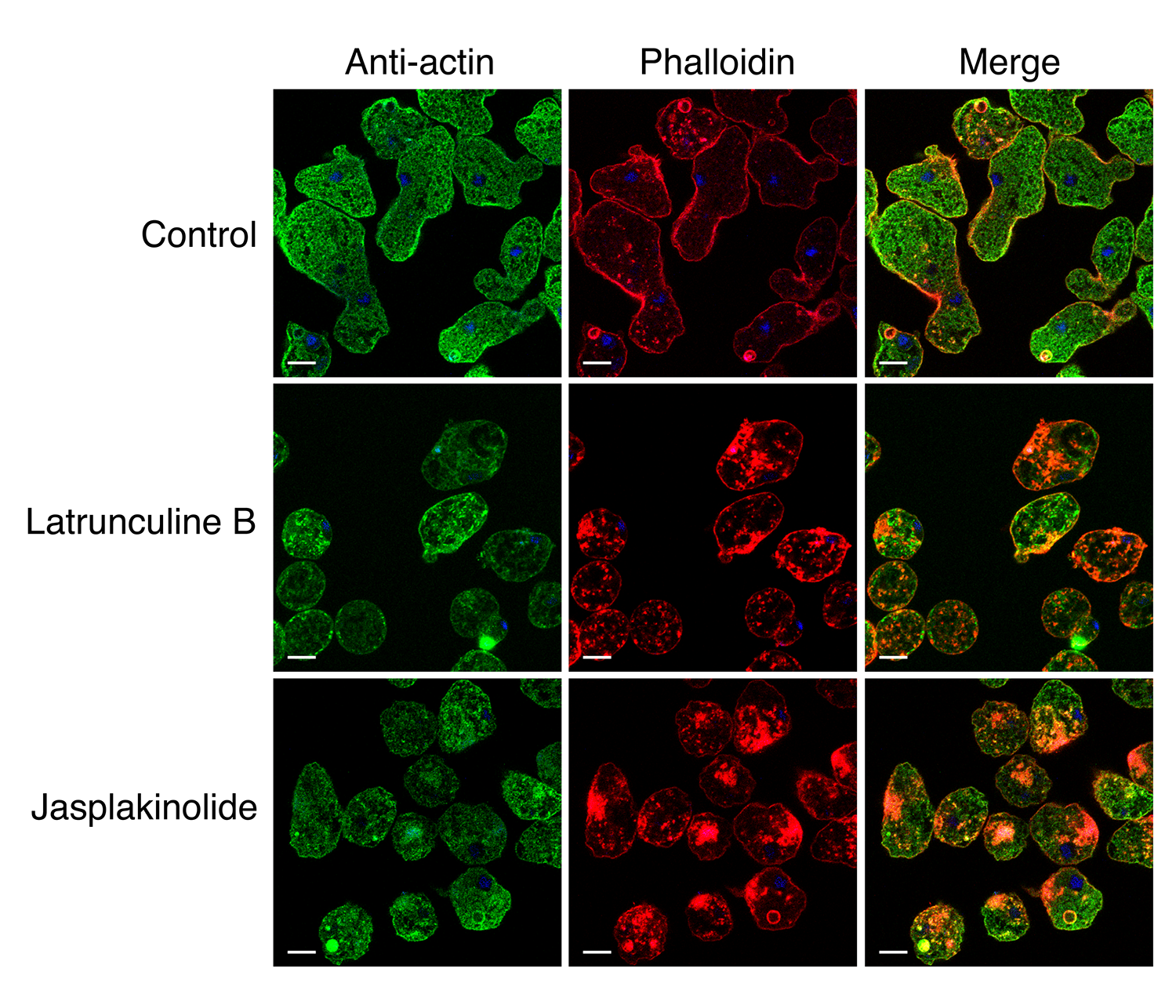

Supplement: Supplemental Figure 1 — E. histolytica actin-rich structures belong to the actin-rich cytoskeleton. Confocal image micrographs from Z-Stack show G-actin (in red, detected by anti-actin antibody) and F-actin (in green, detected by fluorescent phalloidin). Scale bar: 10 μm. (A) Wild-type trophozoites. (B) Trophozoites treated with latrunculin B or jasplakinolide. Scale bar: 10 μm. Treated amoebae displayed an aggregation of actin filaments and adopted a spherical shape, due to the inhibition of actin cytoskeleton dynamics. [file Image_1.TIF]

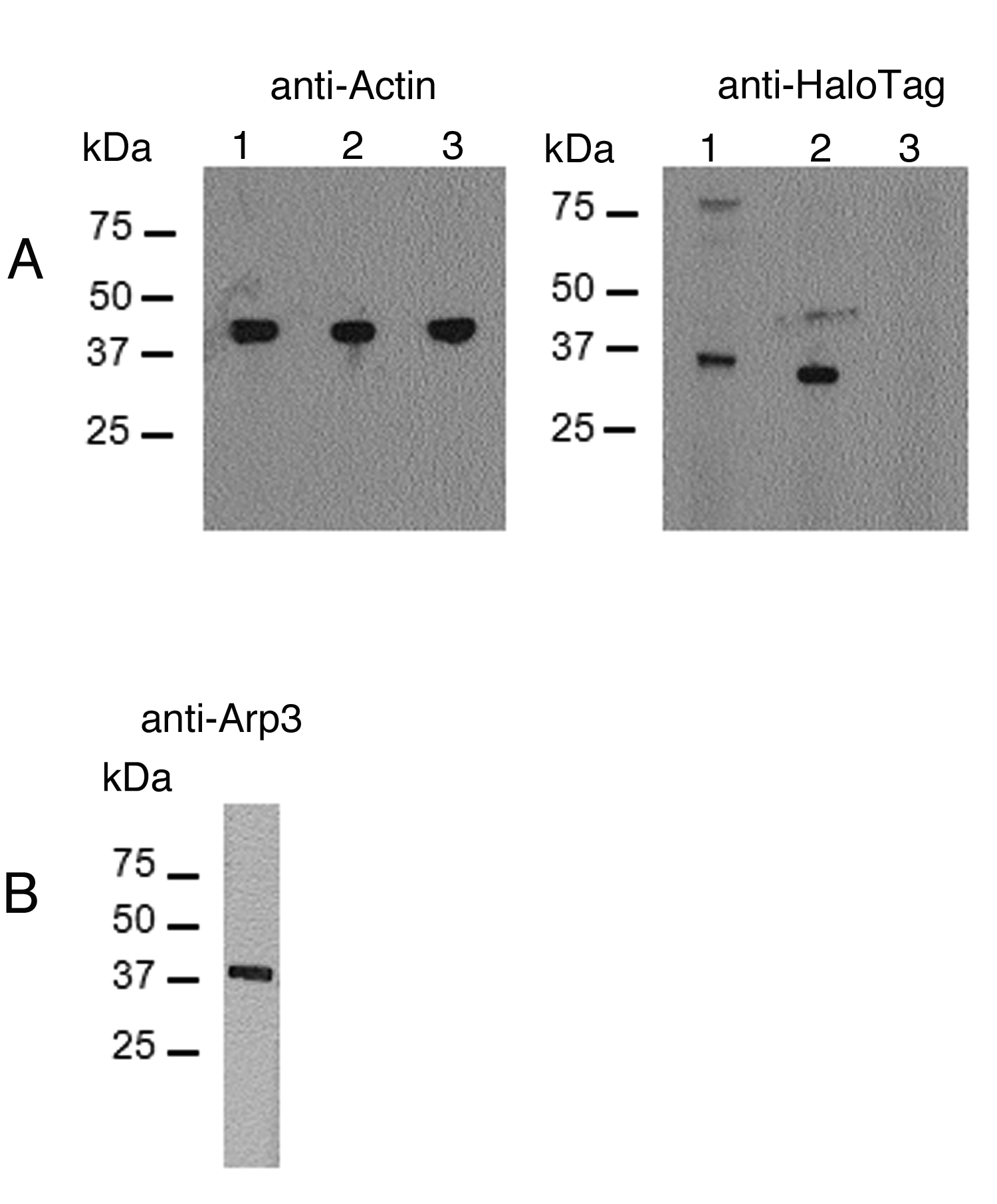

Supplement: Supplemental Figure 2 — Expression of actin-HaloTag in E. histolytica trophozoites. A sample of amoebic extracts (equivalent to 80,000 cells) was resolved by SDS-PAGE and immunoblotted as described in the Material and Methods section. (A) Electrophoretic analysis of proteins from transfected and wild-type amoebae, for the detection of actin and actin-HaloTag. Actin (WT, 42 kDa), HaloTag (H, 34 kDa), and actin-HaloTag (AH, 76 kDa) were detected in the respective protein extracts. As expected, only endogenous actin was detected in both transfected and wild-type amoebae. The anti-HaloTag antibody detected an additional band (at roughly 30 kDa) in the actin-HaloTag protein extract; this may reflect degradation of the fusion protein. Line1 corresponds to Actin-HaloTag cells; line 2 is HaloTag cells and line3 is wild-type amoeba. (B) Electrophoretic analysis of proteins from wild-type amoeba for the detection of Arp3. A single band was seen at 47 kDa. [file Image_2.TIF]
