# Supplementary material for: Morphodynamics of the Actin-Rich Cytoskeleton in Entamoeba histolytica
Source: Front Cell Infect Microbiol. 2018 May 29;8:179. doi: 10.3389/fcimb.2018.00179 (PMC5986921; doi:10.3389/fcimb.2018.00179)
Supplement: Supplemental Datasheet 1 — Nucleotide sequence analysis of actin-encoding genes in E. histolytica HM1:IMSS strain. The DNA sequences were taken from AmoebaDB and aligned using CLUSTAL. The document indicate identity (stars) and homology (double dots) between the DNA sequences in coding regions (A) and promoter regions (150 bp upstream of the ATG initiation codon) (B). [file Data_Sheet_1.DOCX]

**Manich et al., 2018. Supplemental DOCUMENT 1.**

**CLUSTAL O(1.2.4) multiple sequence alignment**

**A. Alignment of DNA sequences corresponding to actin genes from *Entamoeba histolytica***

Percent Identity Matrix - created by Clustal2.1

1: EHI_182900 **100.00** 98.70 99.38 98.62 98.45 93.18 99.29

2: EHI_159150 98.70 **100.00** 99.47 99.40 99.05 98.36 99.20

3: EHI_142730 99.38 99.47 **100.00** 99.38 99.03 99.56 99.56

4: EHI_126190 98.62 99.40 99.38 **100.00** 98.96 98.27 99.47

5: EHI_140120 98.45 99.05 99.03 98.96 **100.00** 98.11 99.29

6: EHI_107290 93.18 98.36 99.56 98.27 98.11 **100.00** 99.47

7: EHI_163750 99.29 99.20 99.56 99.47 99.29 99.47 **100.00**

EHI_182900 ATGGGAGACGAAGAAGTTCAAGCACTTGTTGTAGATAATGGATCAGGAATGTGTAAAGC**T**

EHI_159150 ATGGGAGACGAAGAAGTTCAAGCACTTGTTGTAGATAATGGATCAGGAATGTGTAAAGC**C**

EHI_142730 ATGGGAGACGAAGAAGTTCAAGCACTTGTTGTAGATAATGGATCAGGAATGTGTAAAGC**T**

EHI_126190 ATGGGAGACGAAGAAGTTCAAGCACTTGTTGTAGATAATGGATCAGGAATGTGTAAAGC**C**

EHI_140120 ATGGGAGACGAAGAAGTTCAAGCACTTGTTGTAGATAATGGATCAGGAATGTGTAAAGC**C**

EHI_107290 ATGGGAGACGAAGAAGTTCAAGCACTTGTTGTAGATAATGGATCAGGAATGTGTAAAGC**C**

EHI_163750 ATGGGAGACGAAGAAGTTCAAGCACTTGTTGTAGATAATGGATCAGGAATGTGTAAAGC**T**

***********************************************************

EHI_182900 GGATTTGCTGGTGATGATGCACCAAGAGCTGTATTCCCATCCATTGTTGGTAGACCAAGA

EHI_159150 GGATTTGCTGGTGATGATGCACCAAGAGCTGTATTCCCATCCATTGTTGGTAGACCAAGA

EHI_142730 GGATTTGCTGGTGATGATGCACCAAGAGCTGTATTCCCATCCATTGTTGGTAGACCAAGA

EHI_126190 GGATTTGCTGGTGATGATGCACCAAGAGCTGTATTCCCATCCATTGTTGGTAGACCAAGA

EHI_140120 GGATTTGCTGGTGATGATGCACCAAGAGCTGTATTCCCATCCATTGTTGGTAGACCAAGA

EHI_107290 GGATTTGCTGGTGATGATGCACCAAGAGCTGTATTCCCATCCATTGTTGGTAGACCAAGA

EHI_163750 GGATTTGCTGGTGATGATGCACCAAGAGCTGTATTCCCATCCATTGTTGGTAGACCAAGA

************************************************************

EHI_182900 CACGTTTCAGTTATGGCTGGTATGGGTCAAAA**A**GATGCTTATGTTGGAGATGAAGCTCAA

EHI_159150 CACGTTTCAGTTATGGCTGGTATGGGTCAAAA**G**GATGCTTATGTCGGAGATGAAGCTCAA

EHI_142730 CACGTTTCAGTTATGGCTGGTATGGGTCAAAA**G**GATGCTTATGTTGGAGATGAAGCTCAA

EHI_126190 CACGTTTCAGTTATGGCTGGTATGGGTCAAAA**G**GATGCTTATGTTGGAGATGAAGCTCAA

EHI_140120 CACGTTTCAGTTATGGCTGGTATGGGTCAAAA**G**GATGCTTATGTCGGAGATGAAGCTCAA

EHI_107290 CACGTTTCAGTTATGGCTGGTATGGGTCAAAA**G**GATGCTTATGTTGGAGATGAAGCTCAA

EHI_163750 CACGTTTCAGTTATGGCTGGTATGGGTCAAAA**G**GATGCTTATGTTGGAGATGAAGCTCAA

**********************************.************* ***************

EHI_182900 TCAAAGAG**A**GGTATTCTTACACTTAAATATCCAATTGAACACGGTATTGTTAACAATTGG

EHI_159150 TCAAAGAG**A**GGTATTCTTACACTTAAATATCCAATTGAACACGGTATTGTTAACAATTGG

EHI_142730 TCAAAGAG**A**GGTATTCTTACACTTAAATATCCAATTGAACACGGTATTGTTAACAATTGG

EHI_126190 TCAAAGAG**G**GGTATTCTTACACTTAAATATCCAATTGAACACGGTATTGTTAACAATTGG

EHI_140120 TCAAAGAG**A**GGTATTCTTACACTTAAATATCCAATTGAACACGGTATTGTTAACAATTGG

EHI_107290 TCAAAGAG**A**GGTATTCTTACACTTAAATATCCAATTGAACACGGTATTGTTAACAATTGG

EHI_163750 TCAAAGAG**A**GGTATTCTTACACTTAAATATCCAATTGAACACGGTATTGTTAACAATTGG

**********.*****************************************************

EHI_182900 GA**T**GATATGGAAAAGATCTGGCATCATACTTTCTATAATGAACTTAGAGTTGCACCAGAA

EHI_159150 GA**C**GATATGGAAAAGATCTGGCATCATACTTTCTATAATGAACTTAGAGTTGCACCAGAA

EHI_142730 GA**C**GATATGGAAAAGATCTGGCATCATACTTTCTATAATGAACTTAGAGTTGCACCAGAA

EHI_126190 GA**C**GATATGGAAAAGATCTGGCATCATACTTTCTATAATGAACTTAGAGTTGCACCAGAA

EHI_140120 GA**C**GATATGGAAAAGATCTGGCATCATACTTTCTATAATGAACTTAGAGTTGCACCAGAA

EHI_107290 GA**C**GATATGGAAAAGATCTGGCATCATACTTTCTATAATGAACTTAGAGTTGCACCAGAA

EHI_163750 GA**C**GATATGGAAAAGATCTGGCATCATACTTTCTATAATGAACTTAGAGTTGCACCAGAA

** *********************************************************

EHI_182900 GAACATCCAGTTCTTTTAACTGAAGCCCCAATGAATCCAAAAGCTAACAGAGAAAAGATG

EHI_159150 GAACATCCAGTTCTTTTAACTGAAGCCCCAATGAATCCAAAAGCTAACAGAGAAAAGATG

EHI_142730 GAACATCCAGTTCTTTTAACTGAAGCCCCAATGAATCCAAAAGCTAACAGAGAAAAGATG

EHI_126190 GAACATCCAGTTCTTTTAACTGAAGCCCCAATGAATCCAAAAGCTAACAGAGAAAAGATG

EHI_140120 GAACATCCAGTTCTTTTAACTGAAGCCCCAATGAATCCAAAAGCTAACAGAGAAAAGATG

EHI_107290 GAACATCCAGTTCTTTTAACTGAAGCCCCAATGAATCCAAAAGCTAACAGAGAAAAGATG

EHI_163750 GAACATCCAGTTCTTTTAACTGAAGCCCCAATGAATCCAAAAGCTAACAGAGAAAAGATG

************************************************************

EHI_182900 ACTCAAATTATGTTTGAAACATTCAACACCCCAGCTATGTATGTTGGAATTCAAGCTGTT

EHI_159150 ACTCAAATTATGTTTGAAACATTCAACACCCCAGCTATGTATGTTGGAATTCAAGCTGTT

EHI_142730 ACTCAAATTATGTTTGAAACATTCAACACCCCAGCTATGTATGTTGGAATTCAAGCTGTT

EHI_126190 ACTCAAATTATGTTTGAAACATTCAACACCCCAGCTATGTATGTTGGAATTCAAGCTGTT

EHI_140120 ACTCAAATTATGTTTGAAACATTCAACACCCCAGCTATGTATGTTGGAATTCAAGCTGTT

EHI_107290 ACTCAAATTATGTTTGAAACATTCAACACCCCAGCTATGTATGTTGGAATTCAAGCTGTT

EHI_163750 ACTCAAATTATGTTTGAAACATTCAACACCCCAGCTATGTATGTTGGAATTCAAGCTGTT

************************************************************

EHI_182900 CTTTCATTATATGCCTCAGGTAGAACTACTGGTATTGTTATGGATTCAGGTGATGGAGTT

EHI_159150 CTTTCATTATATGCCTCAGGTAGAACTACTGGTATTGTTATGGATTCAGGTGATGGAGTT

EHI_142730 CTTTCATTATATGCCTCAGGTAGAACTACTGGTATTGTTATGGATTCAGGTGATGGAGTT

EHI_126190 CTTTCATTATATGCCTCAGGTAGAACTACTGGTATTGTTATGGATTCAGGTGATGGAGTT

EHI_140120 CTTTCATTATATGCCTCAGGTAGAACTACTGGTATTGTTATGGATTCAGGTGATGGAGTT

EHI_107290 CTTTCATTATATGCCTCAGGTAGAACTACTGGTATTGTTATGGATTCAGGTGATGGAGTT

EHI_163750 CTTTCATTATATGCCTCAGGTAGAACTACTGGTATTGTTATGGATTCAGGTGATGGAGTT

************************************************************

EHI_182900 TCACACAC**T**GTCCCAATTTATGAAGGATTCTCACTTCCACATGCTATTCT**T**AGACTTGAT

EHI_159150 TCACACAC**C**GTCCCAATTTATGAAGGATTCTCACTTCCACATGCTATTCT**C**AGACTTGAT

EHI_142730 TCACACAC**T**GTCCCAATTTATGAAGGATTCTCACTTCCACATGCTATTCT**T**AGACTTGAT

EHI_126190 TCACACAC**C**GTCCCAATTTATGAAGGATTCTCACTTCCACATGCTATTCT**C**AGACTTGAT

EHI_140120 TCACACAC**C**GTCCCAATTTATGAAGGATTCTCACTTCCACATGCTATTCT**T**AGACTTGAT

EHI_107290 TCACACAC**T**GTCCCAATTTATGAAGGATTCTCACTTCCACATGCTATTCT**T**AGACTTGAT

EHI_163750 TCACACAC**C**GTCCCAATTTATGAAGGATTCTCACTTCCACATGCTATTCT**T**AGACTTGAT

******** ***************************************** *********

EHI_182900 CTTGCAGGACGTGATCTTACTGATTATCTCATGAAAATCTTAACTGAAAGAGGATATGCT

EHI_159150 CTTGCAGGACGTGATCTTACTGATTATCTCATGAAAATCTTAACTGAAAGAGGATATGCT

EHI_142730 CTTGCAGGACGTGATCTTACTGATTATCTCATGAAAATCTTAACTGAAAGAGGATATGCT

EHI_126190 CTTGCAGGACGTGATCTTACTGATTATCTCATGAAAATCTTAACTGAAAGAGGATATGCA

EHI_140120 CTTGCAGGACGTGATCTTACTGATTATCTCATGAAAATCTTAACTGAAAGAGGATATGCT

EHI_107290 CTTGCAGGACGTGATCTTACTGATTATCTCATGAAAATCTTAACTGAAAGAGGATATGCT

EHI_163750 CTTGCAGGACGTGATCTTACTGATTATCTCATGAAAATCTTAACTGAAAGAGGATATGCT

***********************************************************:

EHI_182900 TTCAC**C**ACTACTGCTGAAAGAGAAATTGTCAGAGATATTAAAGAAAAACTTTGCTATGTT

EHI_159150 TTCAC**T**ACTACTGCTGAAAGAGAAATTGTCAGAGATATTAAAGAAAAACTTTGCTATGTT

EHI_142730 TTCAC**T**ACTACTGCTGAAAGAGAAATTGTCAGAGATATTAAAGAAAAACTTTGCTATGTT

EHI_126190 TTCAC**C**ACTACTGCTGAAAGAGAAATTGTCAGAGATATTAAAGAAAAACTTTGCTATGTT

EHI_140120 TTCAC**C**ACTACTGCTGAAAGAGAAATTGTCAGAGATATTAAAGAAAAACTTTGCTATGTT

EHI_107290 TTCAC**C**ACTACTGCTGAAAGAGAAATTGTCAGAGATATTAAAGAAAAACTTTGCTATGTT

EHI_163750 TTCAC**C**ACTACTGCTGAAAGAGAAATTGTCAGAGATATTAAAGAAAAACTTTGCTATGTT

***** ******************************************************

EHI_182900 GCTGAAGATTTCAATGAAGAAATGCAAAAAGCTGCATCAAGCAGTGAACTTGAAAAGAGC

EHI_159150 GCTGAAGATTTCAATGAAGAAATGCAAAAAGCTGCATCAAGCAGTGAACTTGAAAAGAGC

EHI_142730 GCTGAAGATTTCAATGAAGAAATGCAAAAAGCTGCATCAAGCAGTGAACTTGAAAAGAGC

EHI_126190 GCTGAAGATTTCAATGAAGAAATGCAAAAAGCTGCATCAAGCAGTGAACTTGAAAAGAGC

EHI_140120 GCTGAAGATTTCAATGAAGAAATGCAAAAAGCTGCATCAAGCAGTGAACTTGAAAAGAGC

EHI_107290 GCTGAAGATTTCAATGAAGAAATGCAAAAAGCTGCATCAAGCAGTGAACTTGAAAAGAGC

EHI_163750 GCTGAAGATTTCAATGAAGAAATGCAAAAAGCTGCATCAAGCAGTGAACTTGAAAAGAGC

************************************************************

EHI_182900 TATGAACTTCCAGATGGACAAGTTATTACTGTTGGAAA**C**GAAAGATTCAGATGCCCAGAA

EHI_159150 TATGAACTTCCAGATGGACAAGTTATTACTGTTGGAAA**T**GAAAGATTCAGATGCCCAGAA

EHI_142730 TATGAACTTCCAGATGGACAAGTTATTACTGTTGGAAA**T**GAAAGATTCAGATGCCCAGAA

EHI_126190 TATGAACTTCCAGATGGACAAGTTATTACTGTTGGAAA**T**GAAAGATTCAGATGCCCAGAA

EHI_140120 TATGAACTTCCAGATGGACAAGTTATTACTGTTGGAAA**T**GAAAGATTCAGATGCCCAGAA

EHI_107290 TATGAACTTCCAGATGGACAAGTTATTACTGTTGGAAA**T**GAAAGATTCAGATGCCCAGAA

EHI_163750 TATGAACTTCCAGATGGACAAGTTATTACTGTTGGAAA**T**GAAAGATTCAGATGCCCAGAA

************************************** *********************

EHI_182900 GC**C**CTCTT**C**CAACCATCATTCCTTGGTATGGAATG**C**AATGGTATTCATGAAACTAC**C**TAC

EHI_159150 GC**C**CTCTT**C**CAACCATCATTCCTTGGTATGGAATG**C**AATGGTATTCATGAAACTAC**C**TAC

EHI_142730 GC**C**CTCTT**C**CAACCATCATTCCTTGGTATGGAATG**C**AATGGTATTCATGAAACTAC**C**TAC

EHI_126190 GC**T**CTCTT**C**CAACCATCATTCCTTGGTATGGAATG**C**AATGGTATTCATGAAACTAC**C**TAC

EHI_140120 GC**T**CTCTT**C**CAACCATCATTCCTTGGTATGGAATG**C**AATGGTATTCATGAAACTAC**T**TAC

EHI_107290 GC**C**CTCTT**T**CAACCATCATTCCTTGGTATGGAATG**C**AATGGTATTCATGAAACTAC**C**TAC

EHI_163750 GC**T**CTCTT**C**CAACCATCATTCCTTGGTATGGAATG**T**AATGGTATTCATGAAACTAC**C**TAC

** ***** ************************** ******************** ***

EHI_182900 AATTCAATTATGAAATGTGATGTCGATATCAGAAAGGATCTTTATGGAAATATTGTTCTT

EHI_159150 AATTCAATTATGAAATGTGATGTCGATATCAGAAAGGATCTTTATGGAAATATTGTTCTT

EHI_142730 AATTCAATTATGAAATGTGATGTCGATATCAGAAAGGATCTTTATGGAAATATTGTTCTT

EHI_126190 AATTCAATTATGAAATGTGATGTCGATATCAGAAAGGATCTTTATGGAAATATTGTTCTT

EHI_140120 AATTCAATTATGAAATGTGATGTCGATATCAGAAAGGATCTTTATGGAAATATTGTTCTT

EHI_107290 AATTCAATTATGAAATGTGATGTCGATATCAGAAAGGATCTTTATGGAAATATTGTTCTT

EHI_163750 AATTCAATTATGAAATGTGATGTCGATATCAGAAAGGATCTTTATGGAAATATTGTTCTT

************************************************************

EHI_182900 TCAGGAGGAACATCAATGTATCCAGGTATTAACACCAGACTTGAAAAGGAAATGATTCAA

EHI_159150 TCAGGAGGAACATCAATGTATCCAGGTATTAACACCAGACTTGAAAAGGAAATGATTCAA

EHI_142730 TCAGGAGGAACATCAATGTATCCAGGTATTAACACCAGACTTGAAAAGGAAATGATTCAA

EHI_126190 TCAGGAGGAACATCAATGTATCCAGGTATTAACACCAGACTTGAAAAGGAAATGATTCAA

EHI_140120 TCAGGAGGAACATCAATGTATCCAGGTATTAACACCAGACTTGAAAAGGAAATGATTCAA

EHI_107290 TCAGGAGGAACATCAATGTATCCAGGTATTAACACCAGACTTGAAAAGGAAATGATTCAA

EHI_163750 TCAGGAGGAACATCAATGTATCCAGGTATTAACACCAGACTTGAAAAGGAAATGATTCAA

************************************************************

EHI_182900 TTAGCACCACCAACAATGAAGATTAAGGTTATTGC**A**CCACCAGAAAGAAAATA**C**TC**A**GTT

EHI_159150 TTAGCACCACCAACAATGAAGATTAAGGTTATTGC**A**CCACCAGAAAGAAAATA**T**TC**A**GTC

EHI_142730 TTAGCACCACCAACAATGAAGATTAAGGTTATTGC**C**CCACCAGAAAGAAAATA**C**TC**A**GTC

EHI_126190 TTAGCACCACCAACAATGAAGATTAAGGTTATTGC**C**CCACCAGAAAGAAAATA**C**TC**A**GTC

EHI_140120 TTAGCACCACCAACAATGAAGATTAAGGTTATTGC**A**CCACCAGAAAGAAAATA**C**TC**T**GTT

EHI_107290 TTAGCACCACCAACAATGAAGATTAAGGTTATTGC**A**CCACCAGAAAGAAAATA**C**TC**A**GTC

EHI_163750 TTAGCACCACCAACAATGAAGATTAAGGTTATTGC**C**CCACCAGAAAGAAAATA**C**TC**A**GTC

*************************************.******************* ****:****

EHI_182900 TGGATTGGAGGATCAATTCTTGC**C**TCACTTTCTACATTCCAAAACATGTGGATTACCAAG

EHI_159150 TGGATTGGAGGATCAATTCTTGC**C**TCACTTTCTACATTCCAAAACATGTGGATTACCAAG

EHI_142730 TGGATTGGAGGATCAATTCTTGC**C**TCACTTTCTACATTCCAAAACATGTGGATTACCAAG

EHI_126190 TGGATTGGAGGATCAATTCTTGC**C**TCACTTTCTACATTCCAAAACATGTGGATTACCAAG

EHI_140120 TGGATTGGAGGATCAATTCTTGC**A**TCACTTTCTACATTCCAAAACATGTGGATTACCAAG

EHI_107290 TGGATTGGAGGATCAATTCTTGC**C**TCACTTTCTACATTCCAAAACATGTGGATTACCAAG

EHI_163750 TGGATTGGAGGATCAATTCTTGC**C**TCACTTTCTACATTCCAAAACATGTGGATTACCAAG

*************************.**************************************

EHI_182900 GAAGAATATGATGAATC**T**GGACCAGCTATTGTCCACAGAAAATGCTTCTAA

EHI_159150 GAAGAATATGATGAATC**C**GGACCAGCTATTGTCCACAGAAAATGCTTCTAA

EHI_142730 GAAGAATATGATGAATC**C**GGACCAGCTATTGTCCACAGAAAATGCTTCTAA

EHI_126190 GAAGAATATGATGAATC**C**GGACCAGCTATTGTCCACAGAAAATGCTTCTAA

EHI_140120 GAAGAATATGATGAATC**T**GGACCAGCTATTGTCCACAGAAAATGCTTCTAA

EHI_107290 GAAGAATATGATGAATC**T**GGACCAGCTATTGTCCACAGAAAATGCTTCTAA

EHI_163750 GAAGAATATGATGAATC**T**GGACCAGCTATTGTCCACAGAAAATGCTTCTAA

***************** *********************************

**B. Promoters regions of actin genes in *E. histolytica***

Percent Identity Matrix - created by Clustal2.1

EHI_142730 **100.00** 68.91 61.93 63.08 62.94 62.24 64.29

EHI_107290 68.91 **100.00** 57.73 63.87 62.89 65.10 63.54

EHI_182900 61.93 57.73 **100.00** 64.97 66.00 65.66 66.16

EHI_140120 63.08 63.87 64.97 **100.00** 73.60 74.37 74.87

EHI_163750 62.94 62.89 66.00 73.60 **100.00** 81.91 79.90

EHI_159150 62.24 65.10 65.66 74.37 81.91 **100.00** 93.53

EHI_126190 64.29 63.54 66.16 74.87 79.90 93.53 **100.00**

EHI_142730 ---TAAGAAAAAAAAA-AGAAGTAACGAAGATAAACTTTAAAAAAGGAGTTG---AA-CT

EHI_107290 ---------GTTTTACAATAATTAGAGGAGTTGAACT-AAATAATAGAAGTGATAAAACT

EHI_182900 ---TCTGAAGAAAACTGAAAATAGGAAAAATTCCAAA-AATACAAGGAAGGA------AC

EHI_140120 CAACAAAAAGACTA----TTCATTCAGAACTAATAAA-ACTAAGAAGAGTTG------AA

EHI_163750 ---TCACGATTTTATTGAATAAACAATTAAAAGATCC-TAAAAAAGGAGTTG------AA

EHI_159150 -AATATCGTGTTGTTT--AAATGAACTAAAAAAAGAA-ATAAAAAGGAGGTG------AA

EHI_126190 -TTAGCTAAGAAATTA--AAATGAACTAAAAAAAGAA-ATAAAAAGGAGGTG------AA

* **

EHI_142730 AAGAAATACAACGACAATAAACTTTTAGAATGATAAA--AATAGAAATAACATAATACTA

EHI_107290 TAAAATTGAAACGGTAAGGAACTTTAAAAAGTTTATTTTCTCAACTCTAATGAACTGATA

EHI_182900 TTATAAAATAACGTAAGGAACTATGAAGTTCACCTTCAGTAAAAAAGAAGAAAGACACTA

EHI_140120 CTAAATAAAAAAAGTGAGAACTTTGGAGTTTTCATTATCGTTAACTCCAAACAAAAAGTA

EHI_163750 CTAAATAAAAAAAGTGAGAACTTTGGAGTTTTATAAAAATTAAACTCCAAGAGTCAGGTA

EHI_159150 AAATATAAAAAAAGTGAGAACTTTGAAGTTTAATAACGAGTGAACTCCAAAAGACAGGTA

EHI_126190 AAATATAAAAAAAGTGAGAACTTTGGAGTTTAATAACGAGTGAACTCCAAAAGACAGGTA

* ** * * * * * **

EHI_142730 TTTAAACACT-CTTCAAAAACTATTAAATCATTAATTAATT**ATGGGAGACGAAGAAGTTC**

EHI_107290 TTTAAACAAAAAAACTAAAA-TAAATTATCATTAAATAAAT**ATGGGAGACGAAGAAGTTC**

EHI_182900 TTTAAAGACTGACAAAAACTGAATTGA-ACATTCAATAAAT**ATGGGAGACGAAGAAGTTC**

EHI_140120 TTTAAAGATCATAATGAACTGAATTAAATCATTAATTAATT**ATGGGAGACGAAGAAGTTC**

EHI_163750 TTTAAAGATTGACAAAAACTAAACTAAA-CATTTAATTAAT**ATGGGAGACGAAGAAGTTC**

EHI_159150 TTTAAAGATCATAATAAACTAAACTAAA-CATTCAATTAAT**ATGGGAGACGAAGAAGTTC**

EHI_126190 TTTAAAGATCATAATAAACTAAACTAAA-CATTCAATTAAT**ATGGGAGACGAAGAAGTTC**

****** * ** * **** * * * ********************

**+1**

EHI_142730 AAGCACTTGTTGTAGATAATGGATCAGGAATG

EHI_107290 AAGCACTTGTTGTAGATAATGGATCAGGAATG

EHI_182900 AAGCACTTGTTGTAGATAATGGATCAGGAATG

EHI_140120 AAGCACTTGTTGTAGATAATGGATCAGGAATG

EHI_163750 AAGCACTTGTTGTAGATAATGGATCAGGAATG

EHI_159150 AAGCACTTGTTGTAGATAATGGATCAGGAATG

EHI_126190 AAGCACTTGTTGTAGATAATGGATCAGGAATG

********************************

**Consensus: TATTTAAA**C/G**A**--------**AA**----**A**------**CATT**C/T/A**A**A/T**T**A/T**A**A/T**T ATG**
